# Boronate affinity-based photoactivatable magnetic nanoparticles for the oriented and irreversible conjugation of Fc-fused lectins and antibodies†
†Electronic supplementary information (ESI) available. See DOI: 10.1039/c9sc01613a


**DOI:** 10.1039/c9sc01613a

**Published:** 2019-08-05

**Authors:** Chen-Yo Fan, Yi-Ren Hou, Avijit K. Adak, Juanilita T. Waniwan, Mira Anne C. dela Rosa, Penk Yeir Low, Takashi Angata, Kuo-Chu Hwang, Yu-Ju Chen, Chun-Cheng Lin

**Affiliations:** a Department of Chemistry , National Tsing Hua University , Hsinchu , Taiwan . Email: cclin66@mx.nthu.edu.tw; b Institute of Chemistry , Academia Sinica , Taipei , Taiwan . Email: yjchen@gate.sinica.edu.tw; c Institute of Biological Chemistry , Academia Sinica , Taipei , Taiwan; d Frontier Research Center on Fundamental and Applied Sciences of Matters , Hsinchu , Taiwan; e Department of Medicinal and Applied Chemistry , Kaohsiung Medical University , Kaohsiung , Taiwan

## Abstract

The utilization of immuno-magnetic nanoparticles (MNPs) for the selective capture, enrichment, and separation of specific glycoproteins from complicated biological samples is appealing for the discovery of disease biomarkers. Herein, MNPs were designed and anchored with abundant boronic acid (BA) and photoreactive alkyl diazirine (Diaz) functional groups to obtain permanently tethered Fc-fused Siglec-2 and antiserum amyloid A (SAA) mAb with the assistance of reversible boronate affinity and UV light activation in an orientation-controlled manner. The Siglec-2–Fc-functionalized MNPs showed excellent stability in fetal bovine serum (FBS) and excellent efficiency in the extraction of cell membrane glycoproteins. The anti-SAA mAb-functionalized MNPs maintained active Ab orientation and preserved antigen recognition capability in biological samples. Thus, the BA–Diaz-based strategy holds promise for the immobilization of glycoproteins, such as antibodies, with the original protein binding activity maintained, which can provide better enrichment for the sensitive detection of target proteins.

## Abstract 2

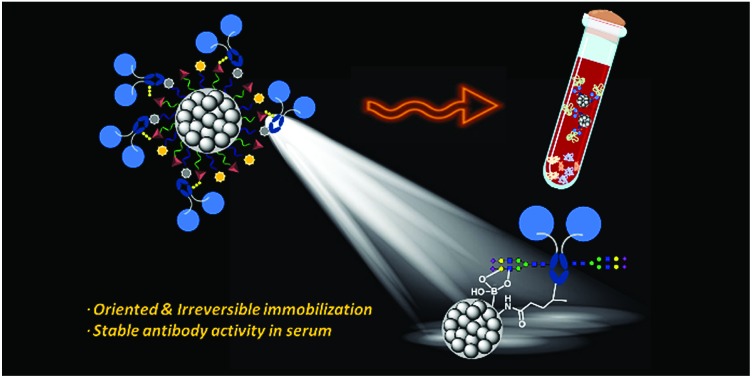
A combination of boronic acid and a photoactivatable diazirine enables oriented conjugation of Fc-fused lectins and antibodies on nanoparticles.

## Introduction

Multifunctional metal nanoparticles (NPs), in particular superparamagnetic iron oxide NPs, have been used as biochemical sensors and biomolecular imaging contrast agents in extensive biomedical applications, including diagnostics, therapeutics, and theranostics.^1^–^3^ A tunable core size, varying from a few up to tens of nanometers, and various shapes (spherical, hexagonal, and cubic) combined with the large surface area to volume ratio of magnetic nanoparticles (MNPs), make them suitable for high-capacity binding to proteins and cells.^4^ The biocompatibility and biodegradability of MNPs featuring unique magnetic and heat dissipation characteristics are central to their successful uses in several *in vitro* and *in vivo* applications.^5^ The utilization of monoclonal antibody (mAb)–MNP nanoconjugates for whole blood purification,^6^ controlling cancer-cell death,^7^ tumor targeting,^8^ neural stem cell extraction,^9^ and the detection of biomarkers^10^–^12^ and bacterial cells^13^ is becoming more common. mAbs offer exquisitely specific and sensitive antigen recognition in such applications, and therefore, strategies for the functionalization of MNP surfaces with Abs are essential for the utilization of MNPs in high-performance immunodiagnostic technologies. To provide better antigen binding activity, a full-length Ab (∼150 kDa, length *ca.* 10 nm)^14^ immobilized on a small surface area of an MNP (113 nm^2^ for 6 nm MNPs) requires oriented conjugation. Additionally, most randomly attached Abs sacrifice antigen recognition abilities, resulting in the production of many nonfunctional Abs on the MNP surface, which significantly decreases the sensitivity and reproducibility of Ab–MNP complexes in immunoaffinity applications. Therefore, it becomes increasingly important to conjugate Abs to MNP surfaces covalently with a controllable and homogeneous orientation.

The nature of MNP surface chemistry promotes the modular and controlled conjugation of biomolecules, especially using amine and carboxyl functionalities.^15^ Proteins adsorbed onto MNP surfaces through hydrophobic and polar interactions in random orientations are not sufficiently stable for many applications. One of the most popular methods for the covalent conjugation of MNPs with Abs involves carbodiimide-based coupling^6^,^9^–^13^ between carboxyl groups on the MNP and the most reactive side chain ε-amines of lysine residues of the Ab, which are positioned at various locations on the surface of the Ab. Despite the lack of Ab modification, this strategy can compromise Ab activity due to potential blockade of the Fab site as a result of attachment to a residue at the Fab site or of nondirectional coupling to the NP surface. Several site-specific conjugation strategies have been proposed to avoid the immobilization of Abs on NPs in random orientations. Chemical modifications through disulfide bond reduction in the hinge region^8^ or Fc *N*-glycan oxidation^16^ providing conjugation sites in the Fc region of Ab have been developed. While the thiol reduction strategy may alter the Ab structure and activity, glycan oxidation requires harsh chemical treatments that may cause Ab crosslinking.17*a* Additionally, oxidation conditions may also affect readily oxidizable amino acid residues such as methionine, tryptophan and tyrosine at different locations of the Ab which could ultimately alter the structure and immunoreactivity of the Ab.17*b* Adaptor proteins such as protein A were used to maintain full biofunctionality through binding to a specific site of the Fc region; thus, the oriented and noncovalent immobilization of Ab on the MNP surface is properly controlled.^7^ However, covalent immobilization requires additional genetic modification of the adaptor protein.^18^ This approach is more expensive and time consuming. Although specific absorption between the Ab and MNP has been proposed for orienting the Ab on the MNP surface by covalent conjugation,^19^ it remains a significant challenge to reliably link unmodified Abs site-specifically to nanoparticle surfaces without impeding their antigen binding capabilities.

Boronic acids (BAs) offer attractive alternative approaches involving common chemical conjugation strategies and provide opportunities for the site-specific attachment of Abs to solid supports.^20^ Recently, reversible covalent bonds between BAs and 1,2-/1,3-*cis*-diols have been implemented in applications involving drug delivery,^21^ protein^22^ and cell^23^ conjugation, and surface immobilization^20^ in addition to their traditional use in the construction of molecular receptors.^24^ Because of the pH-dependent formation/dissociation of boronate ester complexes and advantages such as multiple stable interaction sites and easy magnetic separation, BA-functionalized MNPs have been commonly applied to the specific enrichment of glycoproteins.^25^–^28^ We and others have utilized this orthogonal boronate-mediated bioconjugation for immobilizing mAbs and Fc-fused lectins onto solid supports, including MNPs.^29^–^35^ However, an apparent disadvantage of reversible boronate complexation is that an assay performed in alkaline media may lead to the serum-induced dissociation of some protein-bound complexes on BA-containing surfaces, considerably compromising the assay detection sensitivity under physiological conditions.^36^,^37^ Thus, to prevent protein release, complementary surfaces to which primary Abs can be permanently attached are essential. In this regard, we judiciously combined a BA with the photoaffinity labelling (PAL) reagent trifluoromethylphenyl diazirine (Diaz) for irreversible crosslinking of the constant Fc region of mAbs to planar glass surfaces without affecting the antigen binding affinity, which showed resistance to fouling from biological fluids.^37^ Recently, PAL reagent-functionalized glyco-gold NPs were introduced and described for the enrichment of photocrosslinked lectins.^38^ To fully facilitate the implementation of covalent Ab conjugation in the development of biomolecular assays, further studies are essential to expand the scope of materials that can be surface engineered by UV light activation. Consequently, utilizing a magnetic core unit together with a BA affinity ligand and a photoactivatable group in an integrated platform is feasible for the oriented immobilization of proteins.

Herein, we fabricate a series of BA–alkyl Diaz-functionalized MNPs (BA–Diaz@MNPs) and then covalently attach a Fc-fused Siglec (sialic acid-binding immunoglobulin (Ig)-like lectin), Siglec-2–Fc, or a full-length antiserum amyloid A (SAA) mAb in a highly controllable way for the first time. We show that this boronate-affinity-based oriented and light-induced irreversible fabrication procedure is superior to immobilizing *via* direct random and oriented protein G-based methods. Furthermore, the advantages of oriented and covalently immobilized proteins on MNPs were investigated by the enrichment of Siglec-2-specific membrane-bound glycoproteins as well as the extraction of the SAA human antigen.

## Results and discussion

### Concept of boronate affinity-based photoimmobilization

**Fig. 1:**
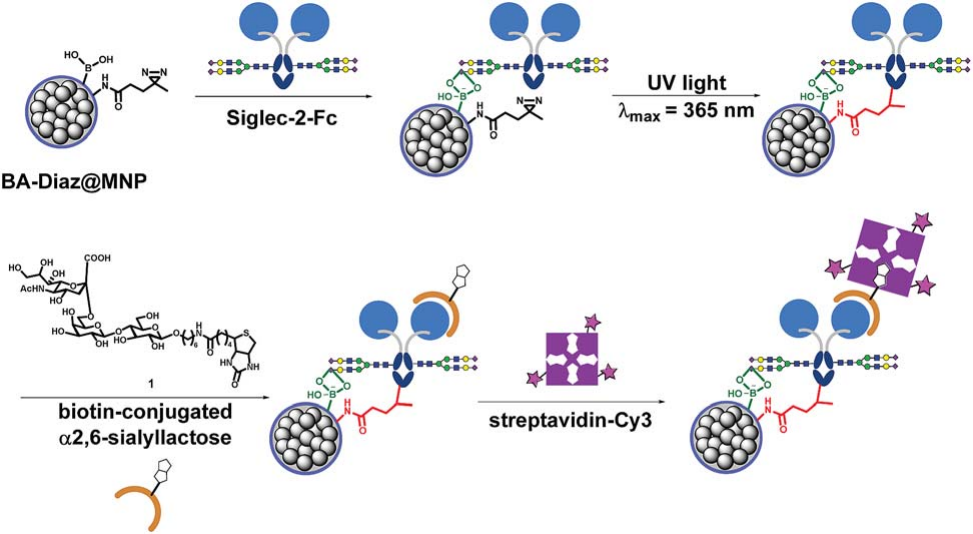
Fabrication of oriented irreversibly Siglec-2–Fc-conjugated MNPs using boronate affinity-based photoimmobilization. BAs associate with the carbohydrate moiety in the Fc region to bring the protein into close proximity, and upon UV irradiation, covalent crosslinking occurs between carbenes generated from Diaz residues and boronated-Fc protein complexes, leading to irreversible and oriented attachment of the glycoprotein to the MNP surface. The functionality of Siglec-2–Fc was tested by incubation with the biotin-conjugated 2,6-sialyllactose ligand followed by fluorescent streptavidin. The protein activity was evaluated by quantifying the dye fluorescence.

Siglec-2 (a mammalian cell surface lectin also known as CD22), an important inhibitory B-lymphocyte (B cell) receptor, was used as the target protein^39^ for irreversible conjugation on MNPs in an oriented manner, as outlined in Fig. 1. A recombinant fusion protein consisting of the human IgG1 Fc fragment fused with the extracellular domain of Siglec-2 (Siglec-2–Fc)^40^ was prepared to facilitate carbohydrate–BA interactions. In the presence of Siglec-2–Fc, the surface BAs on MNPs associate with proteins by targeting the *N*-glycan chain in the constant Fc region (equivalent to Asn297). Targeting the Fc glycan allows the conjugation of the lectin through a site that is distal from the carbohydrate-recognition domains (CRDs) of Siglec-2. This position permits the CRDs of Siglec-2 to be directed upward into the solution, yielding good accessibility for binding to the carbohydrate ligand on the surface of the MNP. Upon exposure to UV light, a covalent adduct is formed by the insertion of reactive carbene species generated from Diaz residues into neighboring C–H or heteroatom-H bonds of the interacting protein, ultimately producing irreversibly tethered Siglec-2–Fc@MNPs. Proteins bound on the MNPs were then detected using a biotinylated 2,6-sialyllactose ligand (**1**),^41^ a known ligand for Siglec-2, followed by visualization with streptavidin-Cy3 with the expectation that an accessible immobilization conformation was maintained. The immobilization technique, being site-selective, can improve the detection sensitivity of the conjugated protein as a result of maximizing the retention of the ligand binding capacity of the protein on the MNP.

### Preparation of functionalized MNPs

**Scheme 1:**
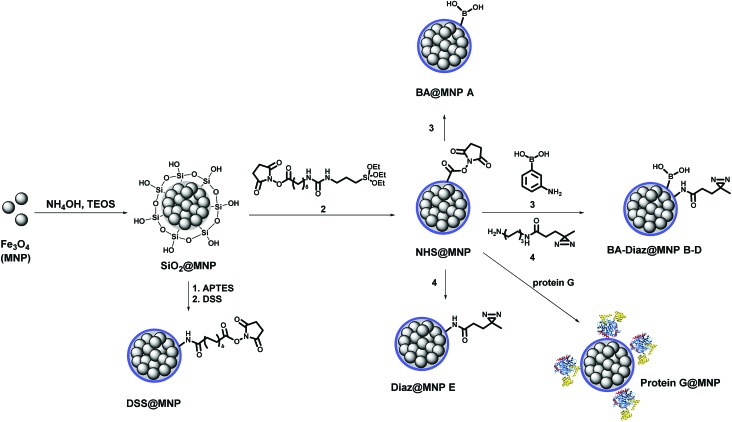
Preparation of functionalized MNPs.

Scheme 1 shows the fabrication of nanoparticles containing an Fe_3_O_4_ magnetic core and their surface modification with BA and Diaz functionalities. The synthesis of the Fe_3_O_4_ core and coating of its surface with tetraethyl orthosilicate (TEOS) were performed according to our previous reports.^35^,^42^ The siloxane surface coating (SiO_2_@MNP) endows the NPs with several important properties, including the ability to attach ligands covalently to the surface, which improves particle resistance to environmental stress, extreme pH, and high ionic strength. The *N*-hydroxysuccinimidyl (NHS) ester-activated MNP, NHS@MNP, was obtained by reacting heterobifunctional siloxane **2** with SiO_2_@MNP. To quantitatively estimate the relevant composition of MNPs upon Siglec-2 immobilization, BA–Diaz@MNPs were designed and synthesized to present five different molar ratios of 3-aminophenylboronic acid (APBA) **3** and Diaz **4** (1 : 0 as BA@MNP **A**; 1 : 10 as BA–Diaz@MNP **B**; 1 : 1 as BA–Diaz@MNP **C**; 10 : 1 as BA–Diaz@MNP **D**; and 0 : 1 as Diaz@MNP **E**). BA@MNP **A** and Diaz@MNP **E**, which lack the photoreactive group and BA affinity head group, respectively, were employed as controls. The photoreactive alkyl Diaz **4** was chosen because of its small size and wide application in PAL experiments.^43^ The NHS esters were readily functionalized through amide condensation with amines of **3** and Diaz **4**, forming mixed layer-protected BA–Diaz@MNP **B–D**. The remaining NHS esters were capped by immersing the conjugated MNPs into a solution containing 2-((2-2-methoxyethoxy)ethoxy)ethanamine (MEE, 40 mM) prior to the protein immobilization step. The BA–Diaz@MNPs were stored at 4 °C and kept away from light for three months without noticeable decomposition. Similarly, protein G was conjugated with NHS@MNPs by amide bond formation followed by deactivation of the remaining NHS with MEE (40 mM) to give protein G@MNPs. DSS@MNP was synthesized by coating SiO_2_@MNP with APTES followed by reaction with the homobifunctional disuccinimidyl suberate (DSS) crosslinker as reported before.^35^

The sizes of the as-prepared Fe_3_O_4_ core and the resulting MNPs were determined by transmission electron microscopy (TEM), as shown in Fig. S1.† The average diameter of the Fe_3_O_4_ core is approximately 6 nm, and the particles are well dispersed in the aqueous solution. After functionalization with APBA/Diaz, the size of BA–Diaz@MNP **C** increased to 15 nm. However, the incorporation of Siglec-2 onto the nanoparticles resulted in the formation of a larger spherical structure with a relatively uniform size of approximately 163 ± 13 nm (Fig. S1(c)†), possibly due to the increased tendency toward partial aggregation between the lectin and BA on the MNP surface.

### Effect of the BA and Diaz ratio on the immobilization of Fc-fused Siglec-2 on BA–Diaz@MNPs

Considering that BAs can form dynamic covalent complexes with *cis*-diols at physiological pH, we first investigated the effect of the ratio of BA affinity ligand to Diaz on the efficiency of Siglec-2–Fc (50 μg mL^–1^) immobilization on MNPs. BA–Diaz@MNPs **B–D** were incubated in phosphate-buffered saline (PBS) (pH 7.4) containing Siglec-2–Fc (100 μL) at 4 °C for 12 h to allow boronate formation. After the removal of unbound proteins by magnetic separation, the Siglec-2–Fc/MNP complexes were exposed to UV irradiation (*λ*_max_ = 365 nm, 16 mW cm^–2^) for 5 min, thus enabling permanent covalent crosslinking onto the MNP surface. The control MNPs, BA@MNP **A** and Diaz@MNP **E**, were also incubated with Siglec-2–Fc (100 μL) in parallel. Prior to Siglec-2 ligand binding, the Siglec-2–Fc@MNPs were further treated with dextran, which is routinely employed for suppressing nonspecific protein accumulation on solid supports^12^,^32^ and for capping unreacted BAs.

Because of the intrinsic fluorescence quenching effect of the Fe_3_O_4_ magnetic core,^44^ especially in small-scale analysis, the activity of immobilized Siglec-2–Fc was indirectly assayed by using an α(2,6)-linked Neu5Ac ligand, Neu5Acα(2,6)Galβ(1,4)Glc (*K*_d_ ∼ 32 μM)^41^ conjugated with biotin (α(2,6)-sialyllactose–biotin, **1**) to interact with the fabricated Siglec-2–Fc@MNPs. The glycan binding activity of Siglec-2 was evaluated by measuring the Cy3 fluorescence signal resulting from dissociation of the compound **1**–streptavidin-Cy3 complex from Siglec-2–Fc@MNP in denaturing solution. After the incubation of streptavidin-Cy3 (10 μg mL^–1^) with the ligand **1**/Siglec-2–Fc@MNP complex for 1 h, the MNPs were repeatedly washed with PBS. Following treatment with the denaturing agent^45^ guanidine hydrochloride (GdnHCl, 6 M) for 1 h at room temperature and magnetic separation, the fluorescence intensity of the supernatant was measured by using a fluorescence plate reader.

**Fig. 2:**
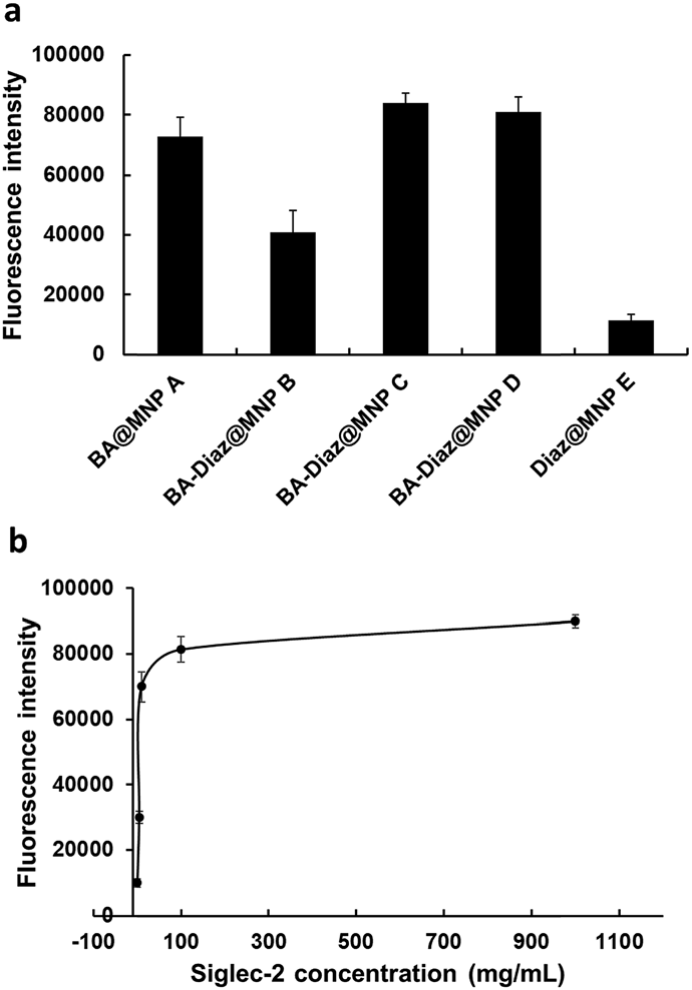
Validation of BA–Diaz-based oriented and irreversible immobilization. (a) Effect of BA and Diaz density on the immobilization of Siglec-2–Fc on the BA–Diaz@MNP surface. (b) Effect of Siglec-2–Fc concentration on the immobilization efficiency of BA–Diaz@MNP **C**. In both cases, fluorescence signals in the supernatant were measured after incubation with α(2,6)-sialyllactose–biotin **1** (100 μM) and streptavidin-Cy3 followed by treatment with GdnHCl. The error bars represent the mean ± SD (standard deviation).

As shown in Fig. 2a, both BA–Diaz@MNP **C** and **D** produced higher fluorescence signals than BA–Diaz@MNP **B,** indicating that larger amounts of Siglec-2–Fc were immobilized on the nanoparticle surface. These results may be attributed to a higher ratio (or an equal amount) of neighboring Diaz residues available on the MNP surface, which could undergo insertion into boronated-protein complexes in higher proportions. Notably, the generated carbenes possess a half-life typically in the nanosecond range, which could be quenched by water in a nonproductive manner, reducing nonspecific protein binding.^42^ While BA@MNP **A** is sufficient to capture appreciable amounts of Siglec-2–Fc (with 0.83-fold fluorescence intensity compared to that of BA–Diaz@MNP **C**), Diaz@MNPs **E** alone shows background absorption. These data clearly suggest that the BA affinity ligand is necessary and that both BA and the photoreactive groups should be spaced in an orderly manner on the surface of the MNP. Although MNP **C** and **D** showed comparable fluorescence signals, we used BA–Diaz@MNP **C** in the following experiments to ensure photo-immobilization efficiency.

### Optimization of UV irradiation time and Siglec-2–Fc concentration for immobilization on BA–Diaz@MNP C

Next, we assayed the effect of UV irradiation time on the photocrosslinking efficiency toward Siglec-2–Fc. Briefly, Siglec-2–Fc (50 μg mL^–1^) was incubated with BA–Diaz@MNP **C** (1.0 mg) at 4 °C for 12 h and then exposed to UV irradiation at 4 °C over a period of time (1–20 min). Our results demonstrated that the fluorescence signals increased in a time-dependent manner, and maximum intensity was achieved with the surface produced under 5 min of UV exposure (Fig. S2†). A longer irradiation time results in a slight decrease in the fluorescence signal, likely causing UV-induced damage to the immobilized proteins.^46^

To investigate the effect of protein concentration on the immobilization efficiency of the BA–Diaz-based fabrication method, 100 μL of Siglec-2–Fc solution with concentrations of 1, 5, 10, 100, and 1000 μg mL^–1^ was used for immobilization on BA–Diaz@MNP **C** (1.0 mg). The results of the binding assays are shown in Fig. 2b. The fluorescence signal reached saturation when the protein concentration was 100 μg mL^–1^. However, when the protein concentration was 5 μg mL^–1^, the resulting Siglec-2@MNPs could still produce a detectable signal in the binding assay. Overall, these results establish an optimal UV exposure time of 5 min for BA–Diaz-based surface immobilization, where we observed the maximum relative Siglec-2–Fc activity on the MNP surface. To lower the consumption of Siglec-2–Fc, in the subsequent experiments, a Siglec-2–Fc solution with a concentration of 50 μg mL^–1^ was used for immobilization to give Siglec-2-BA–Diaz@MNP **C**.

### Assessment of Siglec-2–Fc@MNP stability in complex biological media

**Fig. 3:**
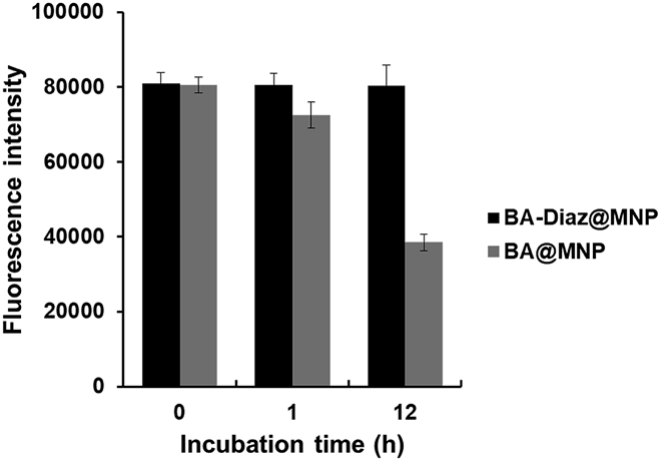
Stability of Siglec-2@MNPs in FBS. Siglec-2@MNPs were fabricated either by using oriented and irreversible conjugation with BA–Diaz@MNP **C** (black) or by reversible boronate diester formation with BA@MNP **A** (gray). The error bars represent the mean ± SD.

Considering the bioreversibility of boron complexation, the stability of Siglec-2–Fc@MNPs in complex biological media was investigated. Proteins that are not covalently bound to nanoparticles should be dissociated under these conditions. Previous reports also indicated that the dynamic interaction of BA/*cis*-diol could be readily interrupted *via* molecular exchange or dissociation in biological fluids such as serum.^36^,^37^ Although the Siglec-2@MNPs fabricated by boronate diester formation using BA@MNP **A** were found to be stable for 1 h of incubation in diluted FBS (FBS : PBS = 1 : 10, pH 7.4), a decrease of approximately 50% in fluorescence intensity (according to the binding assay) was observed after exposure to diluted FBS for 12 h (Fig. 3). Conversely, no apparent changes in the fluorescence intensity were detected when the BA–Diaz method was utilized to prepare Siglec-2–Fc@MNPs under similar conditions, highlighting the importance of irreversible covalent bond formation in the BA–Diaz-based fabrication strategy. Furthermore, sodium dodecyl sulfate–polyacrylamide gel electrophoresis (SDS-PAGE) analysis of Siglec-2-BA–Diaz@MNP **C** showed barely any protein bands corresponding to Siglec-2–Fc in the gel (Fig. S3†). Taken together, these data clearly showed that the proteins are irreversibly linked to the MNPs and are thus suitable for applications under physiological conditions.

### Superiority of the BA–Diaz method for Siglec-2–Fc immobilization

The effect of the immobilization methods used on the surface protein activity was also evaluated. For comparison, 1 mg of each of BA–Diaz@MNP **C**, Protein G@MNP, and NHS@MNP was functionalized with Siglec-2–Fc (50 μg mL^–1^) utilizing three strategies: oriented and covalent boronate diester formation, oriented and noncovalent protein G interaction, and random amide bond formation, respectively. The activity of Siglec-2–Fc on each MNP surface was estimated by a previously described binding assay. As shown in Fig. S4,† the Siglec-2–Fc on BA–Diaz@MNP **C** showed approximately 2.7- and 9.1-fold higher fluorescence signals than protein G@MNPs and NHS@MNPs, respectively. These observations are attributable to the higher protein immobilization efficiency of the BA-containing surface as a result of the higher density distribution of small-sized BA affinity ligands, as reported previously.^32^ Our study suggests that utilization of the current fabrication method provided improved controllability of the site-selective and irreversible attachment of Siglec-2–Fc *via* BA–Diaz residues with uniform orientation and thus yielded more stable and active Siglec-2–Fc on the MNP surface than other methods commonly employed for protein immobilization on nanoparticles.

### Application of Siglec-2-BA–Diaz@MNP **C** for Siglec-2 interacting protein enrichment from BJAB cells

It is known that the non-reducing end NeuAcα2-6Gal in glycoproteins serves as the carbohydrate ligand for Siglec-2,^47^ and the interactions between them modulate B cell receptor signaling.^48^ To understand how these interactions translate into biological outcomes, it is imperative to identify the interacting ligands. The *in situ* identification of Siglec-2 ligands utilizing glycan–protein photocrosslinking^49^ and tyramide proximity labeling^40^ has been reported. In contrast, nanoprobe-based affinity mass spectrometry (NBA-MS)^50^ provides an efficient and straightforward tool for the proteomic study (identification) of interacting glycoprotein ligands. Thus, to further evaluate the advantage afforded by the BA–Diaz immobilization strategy in NBA-MS, Siglec-2–BA–Diaz@MNP **C** was used to enrich interacting membrane proteins from the BJAB human Burkitt lymphoma cell line, which is known to express Siglec-2 ligands.^51^,^52^ To investigate the effect of the immobilization strategy on the resulting Siglec-2 binding activity for interacting protein enrichment, the side chain amines of lysine residues of Siglec-2 were randomly conjugated with activated esters of NHS@MNP to yield Siglec-2–R@MNP. For the negative control, the mutant Siglec-2 (R120A), which is deficient in ligand recognition, was also immobilized on NHS@MNP by similar random immobilization to provide Siglec-2-(R120A)–R@MNP.

**Fig. 4:**
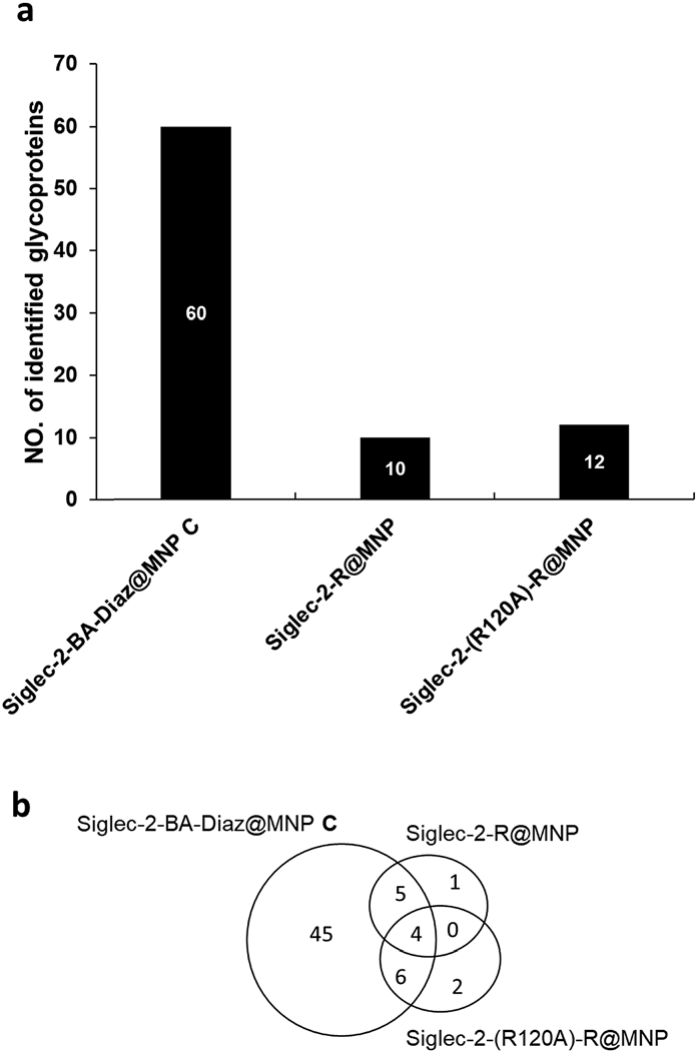
Summary of enriched glycoproteins by Siglec-2–Fc-immobilized MNPs from BJAB cells. For glycoprotein identification, the enriched proteins were trypsinized, and the resulting peptides were analyzed by LC-MS/MS (Synapt G1 Q-ToF MS). (a) Comparison of identified glycoproteins enriched by Siglec-2–BA–Diaz@MNP **C**, Siglec-2–R@MNPs, and Siglec-2-(R120A)–R@MNPs. (b) Overlap of identified glycoproteins from the three MNPs.

To enrich the Siglec-2 interacting proteins, membrane proteins (200 μg) purified from BJAB cells were incubated with each type of Siglec-2@MNP, followed by the tryptic digestion of captured glycoproteins, and then analyzed by LC-MS/MS. Two independent biological replicates and two LC-MS/MS analyses were performed for each experiment. As shown in Fig. 4a, a total of 60, 10, and 12 glycoproteins were identified by Siglec–2-BA–Diaz@MNP **C**, Siglec-2–R@MNP, and Siglec-2-(R120A)–R@MNP, respectively. The oriented and covalently bound Siglec-2–BA–Diaz@MNP **C** gave the highest number of candidate ligands identified, providing superior enrichment compared to the randomly conjugated particles, Siglec-2-R@MNP. We further filtered the potential Siglec-2 ligands using three criteria: (1) inclusion of proteins identified in at least 3 LC-MS/MS runs; (2) inclusion of proteins annotated as glycoproteins in UniProt databases; and (3) exclusion of non-specific binding proteins by removing overlapping proteins in control MNPs. As shown in Fig. 4a and Table S1,† 45 proteins were selected as potential Siglec-2 ligands. The glycoproteins captured by the negative control Siglec-2-(R120A)–R@MNP represent nonspecific interaction. The overlapping proteins obtained by using different MNPs indicate that randomly immobilized Siglec-2–R@MNP may maintain only minimum specific binding activity (overlap with Siglec-2–BA–Diaz@MNP **C**) while oriented Siglec-2–BA–Diaz@MNP **C** can also capture a limited number of proteins, possibly through protein–protein interactions^53^,^54^ (overlap with Siglec-2-(R120A)–R@MNP).

**Fig. 5:**
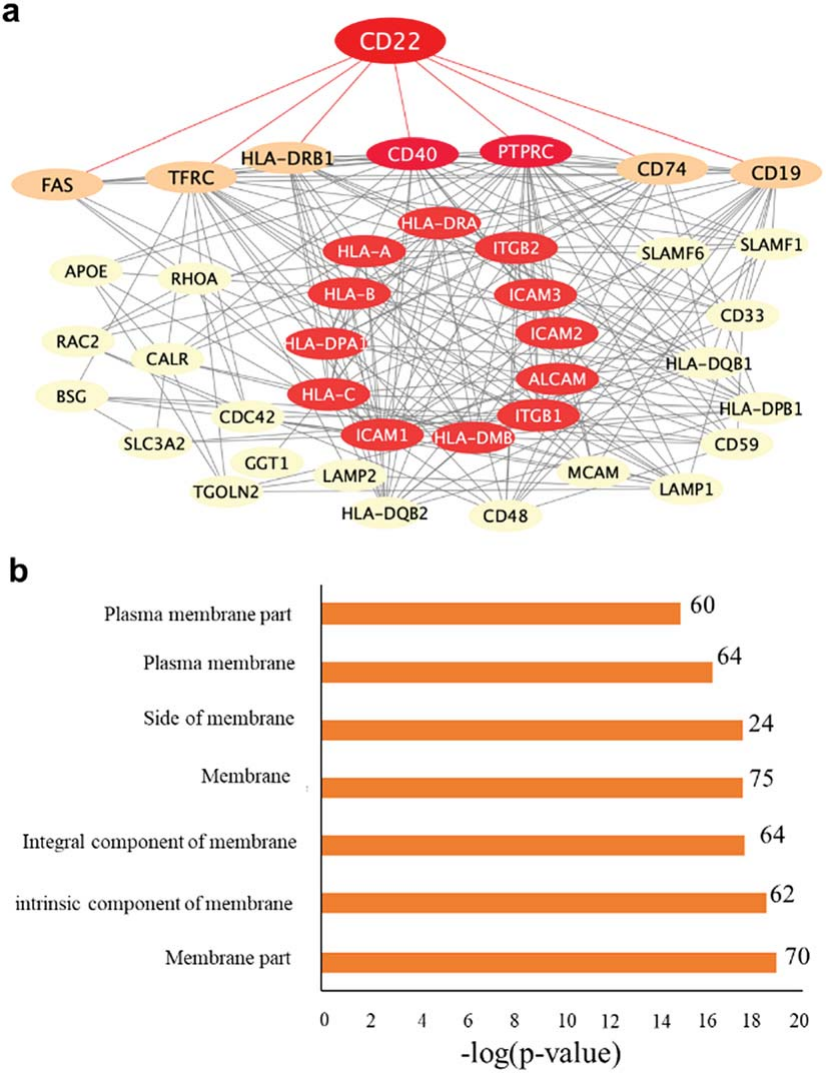
Protein–protein interaction analysis of Siglec-2 (CD22)-bound proteins by using the STRING interaction database. (a) Proteins are represented as nodes. The disconnected nodes and low-*p*-value interactions were filtered out, and a median interaction score of 0.4 was used. Primary and secondary Siglec-2 interacting proteins are shown. Proteins found to have direct interaction with Siglec-2 are connected with red lines. The nodes in red are proteins involved in cell adhesion molecules (CAMs) based on KEGG pathways obtained by STRING analysis. (b) Cellular localization analysis by using STRING also confidently identifies most proteins in the network as membrane proteins.

To further evaluate the effect of the fabrication method on the immobilized protein activity, we purified them again using covalently conjugated Siglec-2–BA–Diaz@MNP **C** and Siglec-2–BA–Diaz–DSS@MNP (obtained by the conjugation of BA–Diaz (1 : 1) to DSS@MNP). A Thermo LTQ Orbitrap Velos mass spectrometer was used for the more sensitive analysis and improved identification of enriched proteins. The potential protein–protein interactions and networks of enriched glycoproteins identified by both particles were annotated by STRING^55^ protein–protein interaction networks and redrawn using Cytoscape. As shown in Fig. 5a, the enriched primary and secondary Siglec-2 (CD22) interactors are highly connected. Proteins directly connected to Siglec-2 include the B cell-specific antigens (tumor necrosis factor receptor superfamily member 5 (CD40) and B-lymphocyte antigen CD19 (CD19)), receptor-type tyrosine-protein phosphatase C (PTPRC), HLA class II histocompatibility antigen gamma chain (CD74), HLA class II histocompatibility antigen (HLA-DRB1), tumor necrosis factor receptor superfamily member 6 (FAS), and transferrin receptor protein (TFRC). Bioinformatics analysis by using STRING according to biological processes indicated the involvement of 46 proteins in the immune system process (FDR = 2.86 × 10^–18^). KEGG pathway analysis also indicated that enriched glycoproteins were tightly associated with cell adhesion molecules (FDR = 7.56 × 10^–21^), identifying 15 proteins as primary or secondary interactors of Siglec-2. Taken together, the bioinformatics analysis revealed a molecular network and clarified the roles of those enriched glycoproteins. Fig. 5b also shows that according to cellular localization analysis by using STRING, a significant number of genes (proteins) in the network are identified with high confidence as membrane proteins.

The lists of identified proteins bound by Siglec-2–BA–Diaz@MNP **C** and the control MNP are shown in Tables S1 and S2.† Human IgG was excluded from the list since the IgG-derived peptides are attributed to the carry-over of the Siglec-2–Fc fusion protein immobilized on the MNPs. On the other hand, Siglec-2 was considered a candidate ligand owing to the absence of peptide fragments in the Siglec-2–Fc fusion protein. It is also noteworthy that there is a significant overlap of identified ligands using the Siglec-2@MNP-based approach and previously reported tyramide labeling and glycan–protein photocrosslinking approaches.^40^,^49^

### Application of anti-SAA mAb–BA–Diaz@MNP to the extraction of acute-phase protein SAA from serum

To demonstrate the generality of the BA–Diaz-based method for glycoprotein/antibody immobilization, anti-SAA mAb was immobilized on BA–Diaz@MNP **C**. SAA is known to be an acute-phase plasma protein implicated in innate immunity and is upregulated in the inflammatory process.^56^ The circulating concentrations of SAA in individuals likely reflect several disease states (inflammation) and are also considered a candidate prognostic marker in late-onset sepsis.^57^ Thus, the sensitive detection of SAA in serum may provide a potential diagnostic for inflammation.

To this end, we first examined the Ab immobilization efficiency on BA–Diaz@MNP **C** at two different pH levels to give anti-SAA mAb–BA–Diaz@MNP **C**. A solution of 25 μL anti-SAA mAb (4 μg μL^–1^) in PBS buffer (10 mM, pH 7.4) was incubated with 1 mg of BA–Diaz@MNP **C** at 4 °C for 12 h. After incubation and magnetic separation followed by UV irradiation, approximately 25 μg mg^–1^ (Ab/MNPs) Ab was immobilized as measured by the bicinchoninic acid (BCA) assay (Fig. S5†). When the pH of the incubation solution was 8.5, the immobilization capacity of BA–Diaz@MNP **C** increased to approximately 50 μg mg^–1^ (Ab/MNP complex). We attributed this difference mainly to the preferential formation of a stable boronated-Ab adduct under slightly basic conditions.^20^,^24^ The initial formation of stable boronate allows more Abs to be photoconjugated upon UV irradiation. For the control, random amide bond formation was used to produce anti-SAA mAb–R@MNP (using NHS@MNP) with a 50 μg Ab/mg MNP complex.

**Fig. 6:**
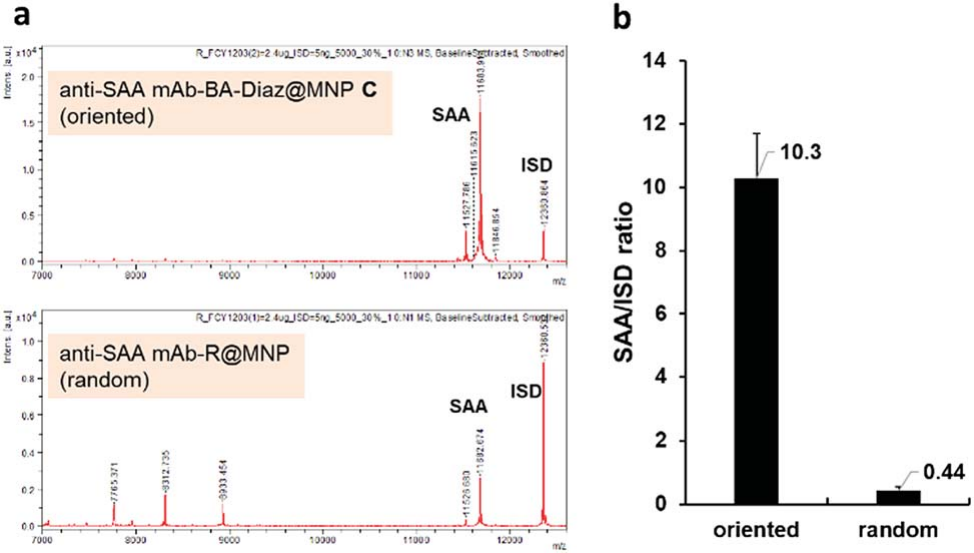
The SAA extraction efficiency of anti-SAA mAb–BA–Diaz@MNP **C** and anti-SAA mAb–R@MNPs. (a) MALDI-TOF MS spectra of the SAA extracted from human serum by anti-SAA mAb–BA–Diaz@MNP **C** (top) and anti-SAA mAb–R@MNPs (bottom). (b) Comparison of SAA enrichment efficiency. The amount of extracted SAA was estimated by comparison to the ratio of SAA/ISD; ISD: internal standard, cytochrome *C*. Error bars denote the SD of duplicate experiments.

To compare the extraction efficiency of Ab@MNPs fabricated by different methods, sera from healthy individuals (3× dilutions in PBS, total volume 60 μL) were incubated with anti-SAA mAb–BA–Diaz@MNP **C** (fabricated at pH 8.5) and anti-SAA mAb–R@MNP (with the same amount of mAb on the surface), respectively, for 1 h at room temperature. After magnetic separation, the relative capture efficiency of the bound antigens was analyzed using the NBA-MS technique. As shown in Fig. 6a, anti-SAA mAb–BA–Diaz@MNP **C** maintains SAA recognition activity and provides better extraction efficiency and specificity for the target antigen at the solid–liquid interface. It is also worth mentioning that the binding of protein interferents is minimal because the immobilization site lies at the Fc moiety, which reduces the steric hindrance to provide a more accessible Fab region.^35^,^58^ Further analysis of the signal intensity revealed that anti-SAA mAb–BA–Diaz@MNP **C** displayed approximately 20-fold higher extraction capacity than randomly conjugated anti-SAA mAb–R@MNP (Fig. 6b). The results indicate that the BA–Diaz-based fabrication strategy achieved a significantly more active immobilized Ab on the MNP surface without decreasing the antigen binding ability.

Despite the promising initial results obtained with the photo-activatable MNP construct developed here, there remain a number of potential areas for improvement. For example, while the extremely short half-life of Diaz leading to a very low crosslinking yield^43^ is already adequate for most applications, it may be possible to obtain even faster and more complete crosslinking by incorporating multivalent PAL reagents into the MNPs. Additionally, since BAs only bind weakly (*K*_d_ = 200 × 10^5^ μM)^24^ to biological polyols, they are not optimized for conjugating many commonly used research Abs. Finally, besides the MNP based applications already examined here, there are a number of additional areas that may readily benefit from using this boronate-affinity based photo-conjugation method. For example, BAs can be used to functionalize microtiter plates with higher Ab densities in an oriented manner or control the amount and location, thereby improving the detection sensitivities of the immunoassays. Moreover, the inherent capability of facile enrichment by magnetic separation has an additional beneficial effect compared with the long-term and high-speed centrifugation by nonmagnetic materials, which often cause entrapment of other background proteins with poor dispersion stability.

## Conclusion

In summary, we have successfully designed and fabricated BA–Diaz-functionalized MNPs that can be utilized to immobilize an Fc-fused lectin and an intact mAb in a more consistently oriented manner. The synergistic effect of the strong affinity of boronate for glycan and light activation yields site-selectively bound proteins linked irreversibly to the MNP surface. Siglec-2–BA–Diaz@MNP **C** showed excellent stability in FBS and exhibited high selectivity for cell membrane glycoproteins with minimum nonspecific binding. Anti-SAA mAb–BA–Diaz@MNP **C** maintains active Ab orientation and preserves antigen recognition capability for the extraction of the target protein from biological samples. To the best of our knowledge, this is the first report of the combination of boronate affinity and photoactivatable MNPs for covalent protein immobilization. Because of the presence of conserved glycan in the Fc region in all mAbs and even in different species, the BA–Diaz-based strategy holds promise for the fabrication of site-selective immunomagnetic nanoconjugates of any given IgG or IgG-like molecules for various immunoaffinity applications. We anticipate that the strategy outlined in this work for MNP functionalization is amenable to the selective enrichment of trace glycoproteins and the characterization of disease biomarkers for clinical diagnosis.

## Experimental section

### Materials and methods

All buffers and solutions were prepared using Millipore water (resistivity equal to 18.2 MΩ cm). Iron(ii) chloride (FeCl_2_·4H_2_O), iron(iii) chloride (FeCl_3_), 3-aminophenyl boronic acid monohydrate (APBA), bovine serum albumin (BSA), dextran (D-1662), and streptavidin-Cy3 conjugate were purchased from Sigma-Aldrich and used as received. Tetraethyl orthosilicate (TEOS) was purchased from Fluka. 25% Ammonia solution and 1-propanol were obtained from Merck. Anti-SAA mAb was purchased from Anogen. 3-(Aminopropyl)trimethoxysilane (APTS) was obtained from Sigma.

### Preparation of NHS@MNPs

Fe_3_O_4_ magnetic nanoparticles (MNPs) were synthesized using FeCl_2_ and FeCl_3_ under basic conditions as described in our previous reports.^35^,^39^ Freshly prepared MNPs (30 mg) were dispersed in 3 mL of 1-propanol (PrOH) and sonicated for 30 min at room temperature (rt). Next, 25% NH_4_OH (0.41 mL) and TEOS (0.1 mL) were added to the above solution, and the mixture was stirred vigorously at 60 °C for 2 h. The resulting black precipitate was collected by using an external magnet and then washed with PrOH (1 mL) three times. Subsequently, the above black MNPs were resuspended in PrOH (3 mL), and then compound **2** (34.5 mg) was added. The resulting mixture was stirred at rt for 6 h. After magnetic separation, the resulting NHS@MNPs were washed with PrOH (1 mL) 3 times to remove excess reagent and stored at 4 °C until use.

### Preparation of BA–Diaz@MNPs

NHS@MNPs (5 mg) were incubated with a solution of 20 mM 3-aminophenyl boronic acid (APBA, **3**) and 20 mM the aliphatic photoaffinity reagent Diaz **4** in DMF with ratios of 1 : 1, 1 : 10, and 10 : 1 (V/V, final volume = 500 μL) at rt for 12 h in a rotary mixer to react with the NHS ester groups on the surface of the MNPs. The MNPs were then separated from the solution by using an external magnet and then washed with DMF followed by deionized water to remove unreacted starting materials. The unreacted NHS esters were then deactivated by immersing the MNPs in a capping solution containing 40 mM 2-((2-2-methoxyethoxy)ethoxy)ethanamine (MEE) in 500 μL of PBS, (pH 7.4) at rt for 3 h. Following separation, the MNPs were washed three times with PBS to provide MNPs **B–D**. The MNPs (**B–D**) were stored at 4 °C in the dark until use. For the preparation of BA@MNP **A** and Diaz@MNP **E**, NHS@MNPs were treated with a solution of **3** (20 mM) and **4** (20 mM) in 500 μL DMF, respectively, at rt for 12 h followed by capping with MEE and then isolated as described before.

### Preparation of protein G@MNP

NHS@MNPs (1 mg) were incubated with 100 μL of protein G (50 μg mL^–1^) in PBS buffer (pH 7.4) at 4 °C in the dark for 12 h in a rotary mixer to react with the NHS ester groups on the surface of the MNPs. The MNPs were then separated from the solution by using an external magnet and then washed with PBS three times to remove unreacted proteins. The unreacted NHS esters were then deactivated by immersing the MNPs in a capping solution containing 40 mM MEE in 500 μL of PBS at rt for 3 h. Following separation, the MNPs were washed three times with PBS to provide protein G@MNP, which was stored at 4 °C in the dark until use.

### BA–Diaz-based photoimmobilization of Siglec-2–Fc on MNPs

One hundred microliters of Siglec-2–Fc solutions in PBS with concentrations of 1, 10, 100, and 1000 μg mL^–1^ were incubated with 1 mg of BA–Diaz@MNP **C** at 4 °C in the dark for 12 h with rotary mixing. Then, the unbound Siglec-2–Fc was washed 3 times with PBS. The Siglec-2–Fc/MNP complexes were then exposed to 365 nm UV light using a Blak-Ray high-intensity UV lamp (*λ*_max_ = 365 nm; 16 mW cm^–2^) at 4 °C for 5 min to covalently immobilize the bound Siglec-2 onto the MNPs, providing Siglec-2–BA–Diaz@MNP **C**. The remaining BAs on the surface were blocked with dextran (100 μL, 100 μM) in water at rt for 2 h prior to binding studies.

### Preparation of DSS@MNPs and oriented photoimmobilization of Siglec-2–Fc on it

Thirty milligrams of freshly prepared Fe_3_O_4_ NPs in PrOH (3 mL) were sonicated for 30 min at rt. Next, 25% NH_4_OH (0.41 mL) and TEOS (0.1 mL) were slowly added to the solution, and the mixture was stirred vigorously at 60 °C for 2 h. APTES (0.1 mL) was then added to the solution, and the mixture was stirred at 60 °C for another 12 h. The MNPs were collected by using a magnet. The residues were washed three times with PrOH (1 mL) to remove excess reagent and then dried under vacuum to obtain aminated Fe_3_O_4_ (NH_2_@MNP) as a black powder. NH_2_@MNPs (1 mg) were suspended in 100 μL of dimethyl sulfoxide (DMSO) and then sonicated for 30 min at rt. Suberic acid bis *N*-hydroxysuccinimide ester (DSS, 5 mg) was added to the solution, and the resulting mixture was stirred at 25 °C for 1 h. The MNPs were separated by using a magnet and then washed with DMSO (100 μL) three times to give DSS@MNPs. The modification of DSS@MNP with BA–Diaz and subsequent conjugation with Siglec-2–Fc were the same as in previous procedures.

### Evaluation of the activity of Siglec-2 on MNPs by the recognition of sialyllactose–biotin ligand and fluorescence analysis

Siglec-2–BA–Diaz@MNPs (1 mg) were added to a solution of α(2,6)-sialyllactose–biotin **1** (100 μL, 100 μM) in PBS, and the mixture was mixed by rotation at rt for 1 h. After incubation, the unbound ligands were washed three times with PBS. A 100 μL solution of streptavidin-Cy3 (10 μg mL^–1^) in PBS was added to the above solution, and the resulting mixture was incubated at rt for 1 h. After washing with PBST (100 μL) three times, the bound streptavidin-Cy3 on the MNPs was released by immersing the complex in 200 μL of 6 M guanidine hydrochloride solution, and the MNPs were removed by using a magnet. The fluorescence intensity of the resulting solution was measured using an ELISA reader (ex/em: 550/570 nm).

### Enrichment of Siglec-2-interacting membrane proteins in BJAB cells by Siglec-2–BA–Diaz@MNP **C** and identification of enriched glycoproteins by LC-MS/MS

Membrane proteins were extracted from BJAB cells.^59^ A total of 200 μg of protein was dissolved with sonication in 500 μL of 6 M urea in PBS. Dissolved membrane proteins were directly added to 150 μg of PBS-washed Siglec-2–Fc conjugated MNPs, and the resulting solution was incubated under vortex for 1 h. The MNPs were collected using a magnet and then washed with incubation buffer and then with 30% acetonitrile in PBS solution. Captured glycoproteins were eluted with 80% acetonitrile and 0.1% (v/v) trifluoroacetic acid in deionized H_2_O. The extracted glycoproteins were dried in a speed vac, resuspended in 200 μL of 6 M urea in 50 mM TEABC, reduced and alkylated with 10 mM DTT and 50 mM IAM, respectively, and diluted to 2 M urea with 50 mM TEABC. The proteins were digested with trypsin in a shaker at 37 °C overnight. The sample was dried in a speed vac, reconstituted in 30 μL of 50 mM ammonium bicarbonate buffer and incubated with PNGase F overnight to obtain deglycopeptides. The sample was desalted by using a C18 ZipTip prior to LC-MS/MS analysis.

### Immunoaffinity purification of SAA using anti-SAA mAb–BA–Diaz@MNP C

Immunoaffinity purification was performed with the aid of Kingfisher™ Magnetic Particle Processors (Thermo Scientific). Anti-SAA mAb–BA–Diaz@MNP **C** (equivalent to 2.4 μg antibody) was added to PBS-diluted human plasma (3× diluted, *V*_T_ = 60 μL). The solution was incubated at rt for 1 h with slow mixing. Then, the MNPs were separated using a magnet, and the residues were washed twice with Tween-Tris-buffered saline (TTBS, 100 μL) and then with deionized water (100 μL). After removal of the water, cytochrome *C* internal standard (1 μL of ISD, 5 ng μL^–1^ in water) and 2 μL of 2′,5′-dihydroxyacetophenone (DHAP) matrix (10 μg μL^–1^ in 50% EtOH/49% H_2_O/1% TFA) were mixed with the MNPs. The supernatant was collected using magnetic separation and then spotted onto a MALDI-MS plate and air dried.

### MALDI-TOF MS analysis

MALDI-TOF-MS spectra were obtained from an ultraFlextreme™ (Bruker, Billerica, MA) equipped with a smartbeam-II™ laser. The MALDI-TOF-MS was operated in positive reflector mode in a mass range of 5000–20 000 Da. A mixture of cytochrome *C* (12 360.97 Da) and myoglobin (16 952.31 Da) was used for external mass calibration. A typical spectrum is obtained by accumulating 5000 shots followed by baseline correction and spectral smoothing using in-house developed software. The SAA-to-ISD ratio was calculated from the sum of the intensities of SAA variants normalized to ISD intensity.

## Conflicts of interest

There are no conflicts of interest to declare.

## Supplementary Material

Supplementary information
