# Correction: Boronate affinity-based photoactivatable magnetic nanoparticles for the oriented and irreversible conjugation of Fc-fused lectins and antibodies

**DOI:** 10.1039/d2sc90049a

**Published:** 2022-03-10

**Authors:** Chen-Yo Fan, Yi-Ren Huo, Avijit K. Adak, Juanilita T. Waniwan, Mira Anne C. dela Rosa, Penk Yeir Low, Takashi Angata, Kuo-Chu Hwang, Yu-Ju Chen, Chun-Cheng Lin

**Affiliations:** Department of Chemistry, National Tsing Hua University Hsinchu Taiwan cclin66@mx.nthu.edu.tw; Institute of Chemistry, Academia Sinica Taipei Taiwan yjchen@gate.sinica.edu.tw; Institute of Biological Chemistry, Academia Sinica Taipei Taiwan; Frontier Research Center on Fundamental and Applied Sciences of Matters Hsinchu Taiwan; Department of Medicinal and Applied Chemistry, Kaohsiung Medical University Kaohsiung Taiwan

## Abstract

Correction for ‘Boronate affinity-based photoactivatable magnetic nanoparticles for the oriented and irreversible conjugation of Fc-fused lectins and antibodies’ by Chen-Yo Fan *et al.*, *Chem. Sci.*, 2019, **10**, 8600–8609, DOI: 10.1039/c9sc01613a.

The authors regret an error in the spelling of Yi-Ren Huo’s name in the original manuscript, the correct spelling is as shown in this Correction article.

The Royal Society of Chemistry apologises for these errors and any consequent inconvenience to authors and readers.

## Supplementary Material

