# Supplementary material for: Boronate affinity-based photoactivatable magnetic nanoparticles for the oriented and irreversible conjugation of Fc-fused lectins and antibodies
Source: Chem Sci. 2019 Aug 5;10(37):8600–9. doi: 10.1039/c9sc01613a (PMC6844280; doi:10.1039/c9sc01613a)
Supplement: Supplementary file 1 [file SC-010-C9SC01613A-s001.pdf]

Electronic Supplementary Material (ESI) for Chemical Science.
This journal is © The Royal Society of Chemistry 2019

SUPPLEMENTARY INFORMATION

Boronate Affinity-based Photoactivatable Magnetic Nanoparticles for Oriented and Irreversible Conjugation of Fc-Fused Lectin and Antibody

Chen-Yo Fan,[a] Yi-Ren Hou,[a] Avijit K. Adak,[a] Juanilita T. Waniwan,[b] Mira Anne C. dela Rosa,[b] Penk-Yeir Low,[c] Takashi Angata,[c] Kuo-Chu Hwang,[a] Yu-Ju Chen,[b,*] Chun-Cheng Lin[a,d*]

[a]Department of Chemistry, National Tsing Hua University, Hsinchu, Taiwan;
[b]Institute of Chemistry and [c]Institute of Biological Chemistry, Academia Sinica, Taipei, Taiwan.
[d]Frontier Research Center on Fundamental and Applied Sciences of Matters

Corresponding Author: Chun-Cheng Lin
Email: cclin66@mx.nthu.edu.tw and yujuchen@gate.sinica.edu.tw

Contents:

Materials and Method....................P3

Synthesis of biotinylated sialoside **1** (**Scheme S1**) P3

Synthesis of N-hydroxysuccinimide ester triethoxysilane **2**, photo-reactive alkyl diazirine **4** (**Scheme S2**) P4

Synthetic procedures and characterization of compounds.................... P4-7

TEM characterization of MNPs (**Figure S1**)....................P7

Effect of UV irradiation time on the immobilization of Siglec-2-Fc on BA-Diaz@MNPs **C** (**Figure S2**).................... P7-8

Non-reducing SDS-PAGE gel analysis of the Siglec-2-Fc@MNPs (**Figure S3**)....................P8

Comparison of relative binding activity of Siglec-2-Fc immobilized on MNPs (**Figure S4**) .P9

Standard curve of BSA quantified by BCA assay (**Figure S5**) P9-10

Liquid Chromatography-Tandem Mass Spectrometry (LC-MS/MS) Analysis LC-MS/MS Method P10-11

Protein Identification and Label Free Quantitation P11

Identified glycoproteins enriched by Siglec-2-BA-Diaz@MNP C and Siglec-2-BA-Diaz-DSS@MNP P12

Glycoproteins and peptides identification analyzed by Thermo LTQ Orbitrap Velos (**Figure S6**) P12

Proteins identified with BA-Diaz@MNP enrichment on BJAB cells (**Table S1**) P13-16

Glycoproteins identified in BJAB cells by Siglec-2-BA-Diaz@MNPs on BA-Diaz-NHS@MNP and BA-Diaz-DSS@MNP (**Table S2**)...P16-20

^{1}H and ^{13}C NMR characterization of compound **1**, **2**, and **4**..P21-26

Reference P27

Materials and Methods

For chemical synthesis, all starting materials and commercially obtained reagents were purchased from Sigma-Aldrich and used as received unless otherwise noted. All reactions were performed in oven-dried glassware under nitrogen atmosphere using dry solvents. ^{1}H and ^{13}C NMR spectra were recorded on Brucker AV-400 spectrometer operating at 400 and 100 MHz, respectively, and were referenced to the solvent used ($CDCl_3$ at δ 7.24 and 77.23, and CD_3OD at δ 3.31 and 49.2 for ^{1}H and ^{13}C, respectively). Chemical shifts (δ) are reported in ppm using the following convention: chemical shift, multiplicity (s = singlet, d = doublet, t = triplet, q = quartet, m = multiplet), integration, and coupling constants (*J*), with *J* reported in Hz. High-resolution mass spectra were recorded under ESI-TOF mass spectroscopy conditions. Analytical thin-layer chromatography (TLC) was performed on pre-coated plates (silica gel 60). Silica gel (E, Merck) was used for flash chromatography. Transmission electron microscopy (TEM) images were obtained by a JEM-2100 electron microscope with an operating voltage of 200 kV.

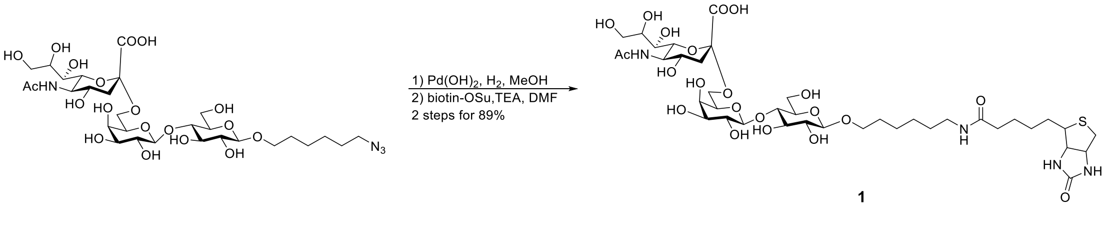

Scheme S1. Synthesis of biotinylated sialoside **1**.

(A)

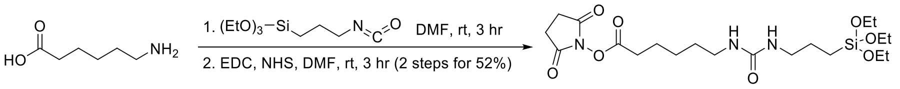

2

(B)

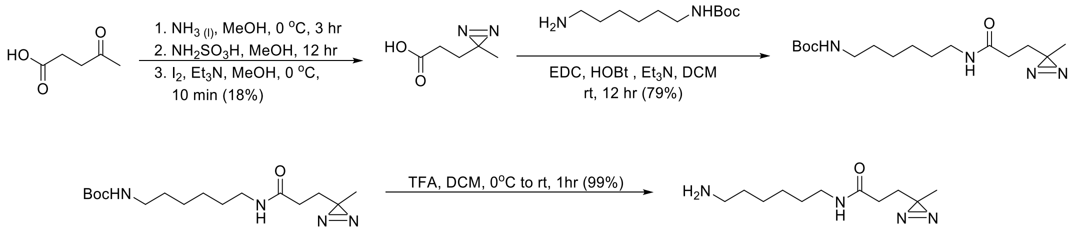

4

Scheme S2. (A) Synthesis of *N*-hydroxysuccinimide ester triethoxysilane **2**. (B) Synthesis of photo-reactive alkyl diazirine **4**.

Compound 1. To a solution of NeuAcα(2,6)Galβ(1,4)Glc[2] (0.026 mmol) in MeOH (0.6 mL) was added palladium hydroxide ($Pd(OH)_2$, 20% Pd content, 4 mg). The mixture was stirred at rt under atmosphere of hydrogen gas for 6 h. The solution was filtered through a pad of Celite. The residue was concentrated to dryness in *vacuo* and the resulting residue was dissolved in DMF (0.6 mL). *N*-Succinimide-biotin (0.039 mmol) was added to the mixture and then stirred at rt for 6 h. The solution was concentrated to dryness in *vacuo* and the resulting residue was purified by C18 reverse phase silica column (Sep-Pak Vac C18 cartridge 20cc/5g 37-55 μm) to give **1** (20 mg, 80% for 2 steps). ^{1}H NMR (400 MHz, D_2O) δ 4.62 (dd, *J* = 4.8, 12.8 Hz, 1H), 4.49 (d, *J* = 8.0 Hz, 1H), 4.44 (d, *J* = 8.0 Hz, 2H), 3.99 (d, *J* = 4.4 Hz, 1H), 3.94-3.54 (m, 18H), 3.33 (q, *J* = 8.8 Hz, 2H), 3.18 (q, *J* = 6.4 Hz, 2H), 3.01 (dd, *J* = 4.8, 12.8 Hz, 1H), 2.79 (d, *J* =

12.8 Hz, 1H), 2.72 (dd, J = 5.2, 12.8 Hz, 1H), 2.26 (t, J = 7.2 Hz, 2H), 2.04 (s, 3H), 1.75 (t, J = 12.8 Hz, 1H), 1.67-1.61 (m, 4H), 1.54 (t, J = 6.8 Hz, 2H), 1.42-1.38 (m, 6H). ^{13}C NMR (100 MHz, D_2O): δ 176.58, 174.93, 173.48, 165.32, 103.24, 101.94, 100.31, 79.70, 74.74, 74.65, 73.71, 72.78, 72.55, 72.39, 71.80, 70.81, 70.54, 68.53, 68.40, 63.55, 62.67, 62.10, 62.02, 60.34, 60.26, 59.35, 55.42, 51.82, 40.12, 39.73, 39.24, 35.92, 35.55, 28.67, 28.24, 28.12, 27.86, 27.70, 25.83. HRMS (ESI) calcd for $C_{39}H_{65}N_4O_{21}S$ [M-H]$^-$ *m/z* 957.3867; found: 957.3862.

Compound 2. A solution of aminocaproic acid (20 mmol) in DMF (30 mL) was added 3-(triethoxysilyl)propyl isocyanate (10 mmol) dropwise under nitrogen. The solution was stirred at rt for 3 h. The mixture was concentrated to dryness *in vacuo* and then diluted with ethyl acetate. The organic layer was washed by H_2O for three times and was dried over $MgSO_4$, filtered, and concentrated. The crude product was then dissolved in DMF (30 mL) followed by adding EDC (15 mmol) and *N*-hydroxysuccinimide (15 mmol). The solution was stirred at rt for 3 h. The mixture was concentrated to dryness *in vacuo* and then diluted with ethyl acetate. The organic layer was washed by H_2O for three times. The organic layer was dried over $MgSO_4$, filtered, and concentrated. The residue was purified by column chromatography on silica gel (MeOH/DCM 1:5) to give **2** (2.53 g, 52% for 2 steps). ^{1}H NMR (400 MHz, $CDCl_3$): δ 3.79 (q, J = 7.2 Hz, 6H), 3.17 (t, J = 6.4 Hz, 2H), 3.12 (t, J = 6.4 Hz, 2H), 2.83 (s,

4H), 2.60 (t, J = 6.4 Hz, 2H), 1.75 (p, J = 7.2 Hz, 2H), 1.60 (q, J = 7.2 Hz, 2H), 1.50 (q, J = 7.2 Hz, 2H), 1.44 (m, 2H), 1.19 (t, J = 7.2 Hz, 9H), 0.60 (dd, J = 8.0, 8.0 Hz, 2H). ^{13}C NMR (100 MHz, $CDCl_3$): δ 169.32 (x2), 168.28, 158.58, 58.07 (x3), 42.51, 39.39, 30.53, 29.24, 25.47, 25.31 (x2), 23.91, 23.38, 17.98 (x3), 7.30. HRMS (ESI) calcd for $C_{20}H_{37}N_3O_8Na$ $[M+Na]^+$ *m/z* 498.2254; found: 498.2248.

Compound 4. A solution of 3-(3-methyl-3H-diaziren-3-yl)propanoic acid[1] (0.5 mmol) in DCM (3 mL) was preactivated with EDC (0.75 mmol), HOBt (0.75 mmol), and trimethylamine (1 mmol) under nitrogen for 30 min. *N*-Boc-1,6-diaminohexane (0.6 mmol) in DCM (3 mL) was then added to the above solution and the resulting solution was stirred at rt for 12 h. The mixture was concentrated to dryness *in vacuo* and then diluted with ethyl acetate. The organic layer was washed with H_2O for three times and was dried over $MgSO_4$, filtered, and concentrated. The crude product was purified by column chromatography on silica gel (MeOH/DCM 1:5). TFA (0.2 mL) was added to a solution of above compound (0.2 mmol) in DCM (1.8 mL) at 0 °C under nitrogen and the resulting solution was stirred at rt for 1 h. The solution was removed and dried *in vacuo* to yield **4** (33 mg, 79% for 2 steps). ^1H NMR (400 MHz, CD_3OD): δ 3.17 (t, J = 6.8 Hz, 2H), 2.91 (t, J = 8.0 Hz, 2H) 2.07 (t, J = 8.0 Hz, 2H), 1.67 (m, 4H), 1.53 (t, J = 6.8 Hz, 2H), 1.40 (t, J = 4.0 Hz, 4H), 1.00 (s, 3H). ^{13}C NMR (100 MHz, CD_3OD): δ 174.68, 40.85, 40.32, 31.70, 31.54, 30.25, 28.65, 27.48, 27.18, 26.51, 19.90. HRMS

(ESI) calcd for $C_{11}H_{23}N_4O$ $[M+H]^+$ *m/z* 227.1872; found: 227.1870.

(a)

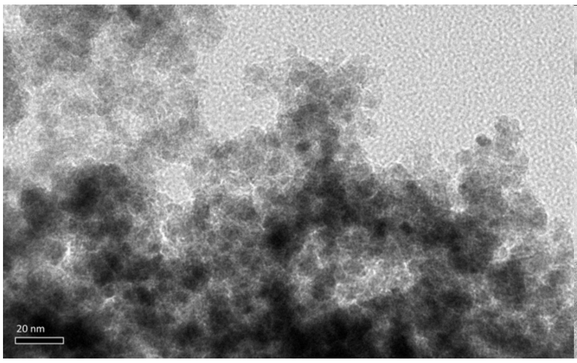

(b)

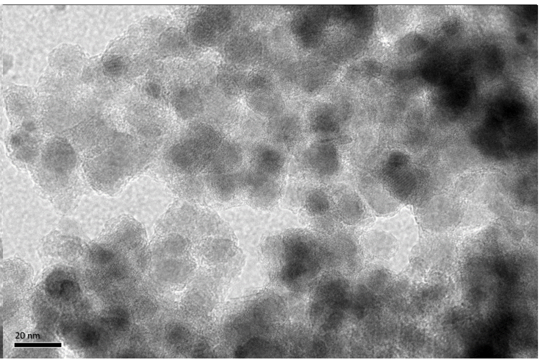

(c)

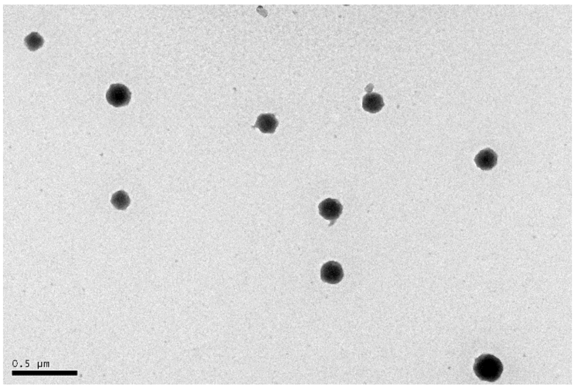

Figure S1. Characterization of MNPs. TEM images of (a) magnetic core Fe_3O_4, (b) BA-Diaz@MNPs **C**, and (c) Siglec-2-BA-Diaz@MNP **C**. The scale bars in TEM images of (a), (b), and (c) are 20, 20, and 500 nm, respectively.

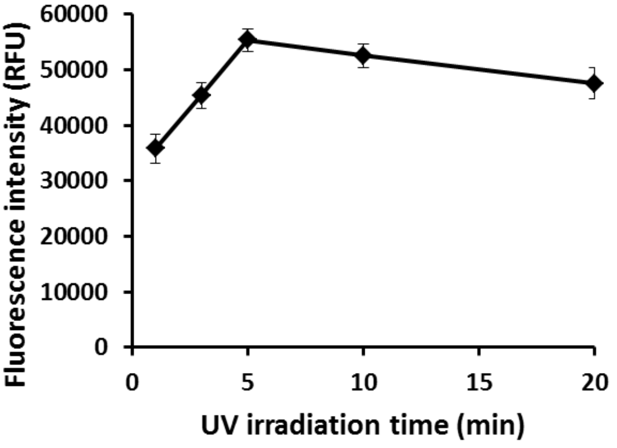

Figure S2. Effect of UV irradiation time on the immobilization of Siglec-2-Fc on BA-Diaz@MNPs **C**. Siglec-2-Fc (50 μg/mL) was incubated on BA-Diaz@MNP **C** followed by UV irradiation for 1, 3, 5, 10 and 20 min, respectively. The fluorescence signals were measured after incubated with biotinylated sialoside **1** and Streptavidin-Cy3 followed by treatment with GdnHCl.

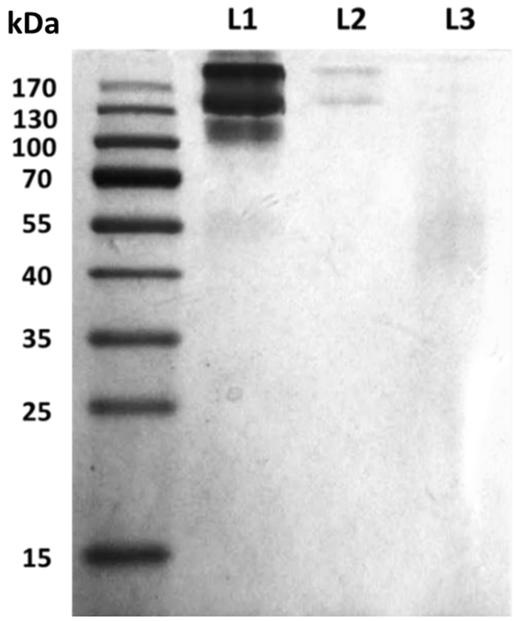

Figure S3. Non-reducing SDS-PAGE gel analysis of the Siglec-2-Fc@MNPs prepared by BA-Diaz-based fabrication strategy showing either traces or no detectable noncovalently attached Siglec-2-Fc on the BA-Diaz@MNP **C**. L1: Siglec-2-Fc (5 μg); L2: Siglec-2-Fc (2 μg) immobilized on BA@MNPs **A** shows partially release of Siglec-2-Fc from MNPs; L3: Siglec-2-Fc (2 μg) immobilized on BA-Diaz@MNP **C**. Protein ladder is shown in left.

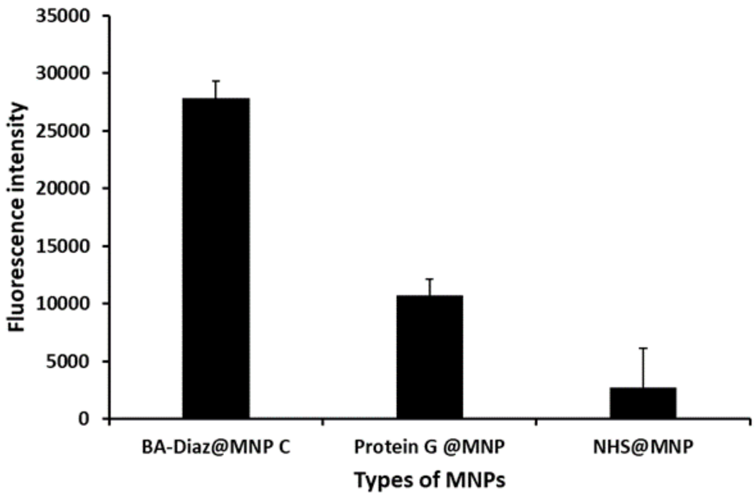

Figure S4. Comparison of relative binding activity of Siglec-2-Fc immobilized on MNPs through oriented and irreversible conjugation utilizing BA-Diaz@MNP **C**, Protein G@MNP, and NHS@MNP. In each case, the same amount of Siglec-2-Fc (50 μg/mL) was used to be immobilized on the same weight of MNPs. Siglec-2-Fc@MNP complexes obtained from different methods were incubated first with biotinylated sialoside **1** and then Streptavidin-Cy3 followed by treatment with GdnHCl. The fluorescence signal of the supernatants were measured.

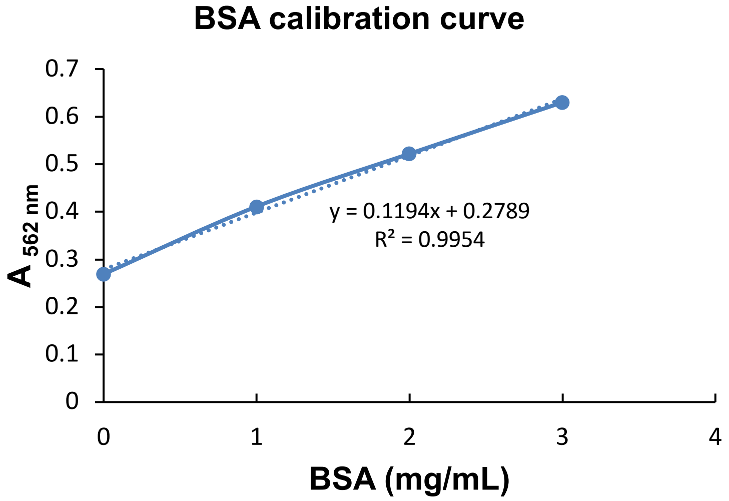

Figure S5. Standard curve of BSA quantified by BCA assay. The linear calibration curve of protein was observed at 562 nm. To quantify the protein amount on MNP, BSA

standard protein and the anti-SAA mAb@MNP samples were prepared and reacted with BCA reagents at 60℃ for 30 min. The quantification curve was obtained by various amount of BSA (0-3 μg/μL). The MNPs were separated by a magnet and the supernatant was used for measurement of absorbance at 562 nm.

Liquid Chromatography-Tandem Mass Spectrometry (LC-MS/MS) Analysis

LC-MS/MS Method. Enriched glycopeptides from BA-Diaz@MNPs and randomly conjugated Siglec-2 MNPs (R@MNPs and R120A@MNPs) were analyzed on a nanoACQUITY UPLC system (Waters, Milford, MA) coupled with a Synapt G1 Q-ToF mass spectrometer (Waters) with a nanospray ion source. The LC mobile phase system consists of water with 0.1% formic acid (buffer A) and acetonitrile with 0.1% formic acid (buffer B). The peptides were eluted with a gradient of 0-80% buffer B (0.1% FA in ACN) for 120 min, operated in ESI-positive V mode. Data acquisition was done by data-dependent acquisition mode to automatically switch between a full MS scan (400-1600 m/z, 0.6 s.) and six MS/MS scans (100-1990 m/z, 0.6 s. for each scan) on the six most intense ions present. For comparison of enrichment efficiency of Siglec-2-BA-Diaz@MNP **C** and Siglec-2-BA-Diaz-DSS@MNP, LC-MS/MS analysis of MNP-enriched glycopeptides was performed on linear trap quadrupole (LTQ)-Orbitrap Velos (Thermo Fisher Scientific, Bremen, Germany) coupled to a nanoACQUITY UPLC System (Waters) through a nanospray interface (Proxeon, Odense, Denmark). The deglycosylated peptides were separated using a segmented gradient for 150-min segmented gradient with the following mobile phases: water with 0.1% formic acid (buffer A) and 2% to 80% acetonitrile with 0.1% formic acid (buffer B) at 500 nL/min flow rate. The electrospray voltage was 1.8 kV, and the ion transfer tube temperature was 300°C. Collision-induced dissociation (CID) mode using a top20 method was used,

and full MS scans were acquired in the Orbitrap mass analyzer over the *m/z* 350–1600 range with resolution 60,000 (*m/z* 400). The target value was 5.00E+05. The twenty-most intense peaks with charge state 2 to 4 were selected for sequencing and fragmented in the ion trap with normalized collision energy of 35%, activation q = 0.25, activation time of 10 ms, and one microscan. The exclusion duration is 60 sec. The target value was 2000 and the isolation width is 2 Da. The maximum allowed ion accumulation times was 500 ms for full scans and 100 ms for CID.

Protein Identification. The raw data were processed using Proteome Discoverer 2.1 (PD2.1; ThermoFisher Scientific) and peptide identification was performed by Mascot search engine (version 2.3.2) and SEQUEST search engine against the Swiss-Prot database (v2015_12, total 20193 sequences from human). Percolator (strict false discovery rate (FDR) of 0.01 and a relaxed FDR of 0.05) was set and the mass tolerance for precursor ion mass was 10 ppm with the fragment ion tolerance as 0.1 Da. The following parameters were used: protease specified as trypsin, maximum missed cleavage set to two, variable modifications selected were methionine oxidation, cysteine alkylation by iodoacetamide (carbamidomethyl), and deamidation at asparagine or glutamine. Peptides were considered identified if their individual ion score was higher than the identity score ($p < 0.05$). A decoy database search against a randomized decoy database created by PD2.1 was performed to evaluate the FDR (<1%) in protein identification. Peptide-spectrum matches (PSMs) with at least high confidence and a strict maximum parsimony principle (target FDR < 0.01) were applied for the protein level. Precursor Ions Area Detector node was used to extract the ion chromatogram for label-free quantitation. The peak area (i.e., area under the curve) of each precursor ion was obtained and the abundance of identified protein was calculated from the top three of all unique and razor peptides in Peptide and Protein Quantifier

node.

Identified glycoproteins enriched by Siglec-2-BA-Diaz@MNP C and Siglec-2-BA-Diaz-DSS@MNP. The enriched proteins, obtained from single biological replicate, were analyzed by the above methods. Using label-free quantification to compare the enriched glycoproteins, 36 proteins showed higher than 5-fold abundance in Siglec-2-BA-Diaz@MNP **C** in comparison with Siglec-2-BA-Diaz-DSS@MNP. The specificities of the materials, calculated as the ratio of glycoproteins over total proteins identified, are 20% and 24% for BA-Diaz@MNP **C** and BA-Diaz-DSS@MNP, respectively. The proteins commonly found from both MNP enrichments were then further analyzed by STRING database to create the CD-22 (Siglec-2) interacting network. For the protein signaling network analysis, the glycoproteins were analyzed using STRING version 11 (http://string-db.org/). Multiple protein names were inputted into String, the organism was set as Homo sapiens, and prediction methods included the following: neighborhood, gene fusion, cooccurrence, coexpression, experiments, databases, textmining and homology. Median confidence (0.4) was used, shown interactors was set as "custom limit=0", and the additional (white) nodes was set as 0. A signaling network composed of predicted or known protein interactions was then obtained and Cytoscape was used to redraw the protein network to show only CD-22 interacting proteins and secondary interactions (interactions with CD-22 interacting proteins).

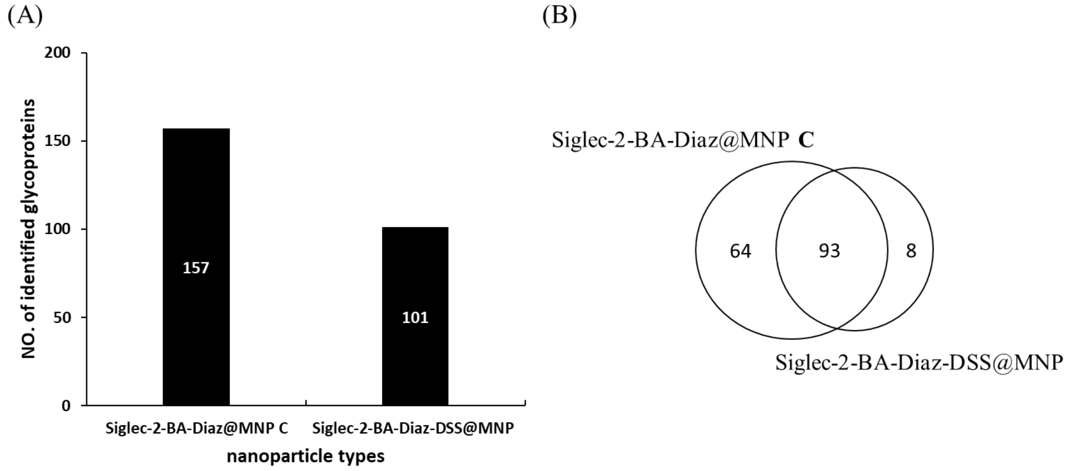

Figure S6. Glycoproteins and peptides identification were analyzed by Thermo LTQ Orbitrap Velos. (a) Identified glycoproteins from Siglec-2-BA-Diaz@MNP **C** and Siglec-2-BA-Diaz-DSS@MNP. (b) Overlap of identified glycoproteins from the two MNPs.

Table S1. Proteins identified with BA-Diaz@MNP enrichment on BJAB cells.

Acc. No.	Gene	Description	Score Mascot	# Unique Peptides	emP AI	Iden-tified in Ref
P20273	CD22 *	B-cell receptor CD22	1778	29	15.24	v
P01903	HLA-DRA*	HLA class II histocompatibility antigen, DR alpha chain	645	11	25.83	v
P04233	CD74	HLA class II histocompatibility antigen gamma chain	589	4	0.97	
Q95IE3	HLA-DRB1*	HLA class II histocompatibility antigen, DRB1-12 beta chain	513	5	12.34	v
Q30134	HLA-DRB1	HLA class II histocompatibility antigen, DRB1-8 beta chain	480	1	5.49	
Q5Y7A7	HLA-DRB1	HLA class II histocompatibility antigen, DRB1-13 beta chain	466	3	14.40	
P01860	IGHG3	Immunoglobulin heavy constant gamma 3	437	1	4.28	
P25705	ATP5A1	ATP synthase subunit alpha, mitochondrial	433	11	1.07	
P30461	HLA-B*	HLA class I histocompatibility antigen, B-13 alpha chain	346	3	1.24	v
P04843	RPN1	Dolichyl-diphosphooligosaccharide--protein glycosyltransferase subunit 1	325	11	1.08	
P79483	HLA-DRB3	HLA class II histocompatibility antigen, DR beta 3 chain	323	2	1.96	
P08195	SLC3A2*	4F2 cell-surface antigen heavy chain	305	11	1.41	v
P04440	HLA-DPB1*	HLA class II histocompatibility antigen, DP beta 1 chain	274	2	0.93	v
Q9GIY3	HLA-DRB1*	HLA class II histocompatibility antigen, DRB1-14 beta chain	258	1	9.00	v
P35613	BSG	Basigin OS=Homo sapiens	229	4	0.59	
P05107	ITGB2*	Integrin beta-2	209	13	1.02	v
P08575	PTPRC*	Receptor-type tyrosine-protein phosphatase C	195	9	0.33	v
P17693	HLA-G	HLA class I histocompatibility antigen, alpha chain G	167	1	0.39	
P04844	RPN2	Dolichyl-diphosphooligosaccharide--protein glycosyltransferase subunit 2	157	4	0.35	
O00754	MAN2B1	Lysosomal alpha-mannosidase	133	3	0.12	
P02748	C9	Complement component C9	131	2	0.16	
P14625	HSP90B1	Endoplasmin OS=Homo sapiens	130	4	0.25	
P01920	HLA-DQB1*	HLA class II histocompatibility antigen, DQ beta 1 chain	123	7	2.16	v
P49755	TMED10	Transmembrane emp24 domain-containing protein 10	108	3	0.70	

P08238	HSP90AB1	Heat shock protein HSP 90-beta	108	6	0.46	
P02042	HBD	Hemoglobin subunit delta	106	1	0.23	
Q12907	LMAN2	Vesicular integral-membrane protein VIP36	94	7	1.68	
Q13724	MOGS	Mannosyl-oligosaccharide glucosidase	77	3	0.23	
P01909	HLA-DQA1	HLA class II histocompatibility antigen, DQ alpha 1 chain	73	4	1.31	
P13473	LAMP2	Lysosome-associated membrane glycoprotein 2	70	2	0.23	
Q9NR96	TLR9	Toll-like receptor 9	66	2	0.08	
P23284	PPIB	Peptidyl-prolyl cis-trans isomerase B	63	1	0.16	
Q14165	MLEC	Malectin	61	1	0.12	
P02786	TFRC*	Transferrin receptor protein 1	58	6	0.31	v
P13987	CD59	CD59 glycoprotein	55	1	0.39	
P54709	ATP1B3	Sodium/potassium-transporting ATPase subunit beta-3	54	3	1.15	
P15153	RAC2	Ras-related C3 botulinum toxin substrate 2	48	3	1.00	
P69905	HBA1	Hemoglobin subunit alpha	45	3	2.16	
Q6NUQ4	TMEM214	Transmembrane protein 214	42	2	0.13	
P11166	SLC2A1	Solute carrier family 2, facilitated glucose transporter member 1	37	1	0.15	
P43307	SSR1	Translocon-associated protein subunit alpha	37	2	0.47	
Q08380	LGALS3BP	Galectin-3-binding protein	37	4	0.32	
P28288	ABCD3	ATP-binding cassette sub-family D member 3	35	2	0.11	
Q13586	STIM1	Stromal interaction molecule 1	35	3	0.20	
P42081	CD86	T-lymphocyte activation antigen CD86	34	1	0.15	
P43308	SSR2	Translocon-associated protein subunit beta	33	2	1.15	
Q96KA5	CLPTM1L	Cleft lip and palate transmembrane protein 1-like protein	32	2	0.39	
P27797	CALR	Calreticulin	32	3	0.30	
P40926	MDH2	Malate dehydrogenase, mitochondrial	32	4	0.45	
Q92896	GLG1	Golgi apparatus protein 1	27	2	0.07	
P08134	RHOC	Rho-related GTP-binding protein RhoC	27	2	0.52	
P20645	M6PR	Cation-dependent mannose-6-phosphate receptor	17	1	0.13	
P62937	PPIA	Peptidyl-prolyl cis-trans isomerase A	0	4	0.93	

P06576	ATP5B	ATP synthase subunit beta, mitochondrial	0	9	0.95	
P60953	CDC42	Cell division control protein 42 homolog	0	2	1.00	
P32970	CD70	CD70 antigen	0	3	0.70	
P11049	CD37*	Leukocyte antigen CD37	0	1	0.17	v
P11717	IGF2R	Cation-independent mannose-6-phosphate receptor	0	3	0.05	
Q15758	SLC1A5*	Neutral amino acid transporter B(0)	0	1	0.11	v

Table S2. Glycoproteins identified in BJAB cells from both Siglec-2-BA-Diaz@MNP **C** and Siglec-2-BA-Diaz-DSS@MNP enrichment.

Accession	Gene	Description	Mascot Score	# unique peptide	emPAI	MW [kDa]
P01857	IGHG1	Immunoglobulin heavy constant gamma 1	12304	8	397.11	36.08
P20273	CD22	B-cell receptor CD22	11589	24	20.98	95.29
P01903	HLA-DRA	HLA class II histocompatibility antigen, DR alpha chain	10884	9	116.88	28.59
Q5Y7A7	HLA-DRB1	HLA class II histocompatibility antigen, DRB1-13 beta chain	6528	2	63.94	29.99
Q95IE3	HLA-DRB1	HLA class II histocompatibility antigen, DRB1-12 beta chain	6371	2	55.23	29.86
P20039	HLA-DRB1	HLA class II histocompatibility antigen, DRB1-11 beta chain	5652	1	41.17	30.14
Q30134	HLA-DRB1	HLA class II histocompatibility antigen, DRB1-8 beta chain	5509	1	26.38	29.99
Q9GIY3	HLA-DRB1	HLA class II histocompatibility antigen, DRB1-14 beta chain	4729	1	14.85	30.12
P79483	HLA-DRB3	HLA class II histocompatibility antigen, DR beta 3 chain	4632	4	28.55	29.94

P04233	CD74	HLA class II histocompatibility antigen gamma chain	4420	10	86.33	33.49
P35613	BSG	Basigin	4287	10	6.94	42.17
P25705	ATP5A1	ATP synthase subunit alpha, mitochondrial	4119	17	2.79	59.71
P05107	ITGB2	Integrin beta-2	4088	29	4.18	84.73
P08195	SLC3A2	4F2 cell-surface antigen heavy chain	3147	18	3.15	67.95
P10316	HLA-A	HLA class I histocompatibility antigen, A-69 alpha chain	3081	7	11.22	40.95
P06576	ATP5B P	ATP synthase subunit beta, mitochondrial	3079	21	10.60	56.53
Q08380	LGALS3BP	Galectin-3-binding protein	2932	17	3.98	65.29
P01920	HLA-DQB1	HLA class II histocompatibility antigen, DQ beta 1 chain	2441	10	12.90	29.97
P10319	HLA-B	HLA class I histocompatibility antigen, B-58 alpha chain	2415	1	3.47	40.31
P30443	HLA-A	HLA class I histocompatibility antigen, A-1 alpha chain	2097	4	5.70	40.82
Q13740	ALCAM	CD166 antigen	1842	12	2.16	65.06
P30504	HLA-C	HLA class I histocompatibility antigen, Cw-4 alpha chain	1829	1	2.79	40.97
P15391	CD19	B-lymphocyte antigen CD19	1567	8	1.22	61.09
P20036	HLA-DPA1	HLA class II histocompatibility antigen, DP alpha 1 chain	1414	5	10.94	29.36
P07602	PSAP	Prosaposin	1347	8	1.11	58.07
Q13586	STIM1	Stromal interaction molecule 1	1322	15	2.57	77.38
P05556	ITGB1	Integrin beta-1	1305	12	1.75	88.36
P13473	LAMP2	Lysosome-associated membrane glycoprotein 2	1239	6	1.31	44.93
P04440	HLA-DPB1	HLA class II histocompatibility antigen, DP beta 1 chain	1195	5	2.73	29.14
Q9BVK6	TMED9	Transmembrane emp24 domain-containing protein 9	1171	7	4.62	27.26

P08575	PTPRC	Receptor-type tyrosine-protein phosphatase C	1134	18	0.94	147.16
P25942	CD40	Tumor necrosis factor receptor superfamily member 5	1093	8	7.48	30.60
P43307	SSR1	Translocon-associated protein subunit alpha	1077	4	3.64	32.22
P08238	HSP90AB1	Heat shock protein HSP 90-beta	1071	10	2.08	83.21
P02786	TFRC	Transferrin receptor protein 1	1021	9	0.50	84.82
P49755	TMED10	Transmembrane emp24 domain-containing protein 10	1014	4	1.89	24.96
P04222	HLA-C	HLA class I histocompatibility antigen, Cw-3 alpha chain	948	1	2.36	40.84
P60953	CDC42	Cell division control protein 42 homolog	890	5	2.98	21.25
Q92542	NCSTN	Nicastrin	833	5	1.02	78.36
P32942	ICAM3	Intercellular adhesion molecule 3	809	6	0.61	59.50
O00461	GOLIM4	Golgi integral membrane protein 4	780	12	1.15	81.83
P04843	RPN1	Dolichyl-diphosphooligosaccharide--protein glycosyltransferase subunit 1	769	15	1.46	68.53
P13987	CD59	CD59 glycoprotein	689	5	12.90	14.17
Q86UP2	KTN1	Kinectin	685	18	0.66	156.18
P13598	ICAM2	Intercellular adhesion molecule 2	675	4	0.90	30.64
P05362	ICAM1	Intercellular adhesion molecule 1	673	7	0.71	57.79
P14625	HSP90B1	Endoplasmin	659	7	0.50	92.41
P23284	PPIB	Peptidyl-prolyl cis-trans isomerase B	623	6	1.74	23.73
Q92896	GLG1	Golgi apparatus protein 1	595	9	0.38	134.46
P02748	C9	Complement component C9	592	2	0.25	63.13
P54709	ATP1B3	Sodium/potassium-transporting ATPase subunit beta-3	554	6	1.93	31.49

P09326	CD48	CD48 antigen	540	5	1.42	27.67
P62937	PPIA	Peptidyl-prolyl cis-trans isomerase A	523	5	1.28	18.00
P11279	LAMP1	Lysosome-associated membrane glycoprotein 1	519	4	0.65	44.85
O43493	TGOLN2	Trans-Golgi network integral membrane protein 2	514	5	0.47	51.08
P19440	GGT1	Gamma-glutamyltranspeptidase 1	509	3	0.33	61.37
P20138	CD33	Myeloid cell surface antigen CD33	506	3	0.47	39.80
Q12907	LMAN2	Vesicular integral-membrane protein VIP36	504	9	3.16	40.20
P05538	HLA-DQB2	HLA class II histocompatibility antigen, DQ beta 2 chain	502	1	0.85	30.37
Q70UQ0	IKBIP	Inhibitor of nuclear factor kappa-B kinase-interacting protein	491	5	0.87	39.29
P61586	RHOA	Transforming protein RhoA	458	7	7.38	21.75
Q9BY67	CADM1	Cell adhesion molecule 1	448	3	0.47	48.48
P27797	CALR	Calreticulin	430	9	2.46	48.11
Q9BUR5	APOO	MICOS complex subunit MIC26	414	3	0.70	22.27
Q96A33	CCDC47	Coiled-coil domain-containing protein 47	412	6	1.02	55.84
Q14165	MLEC	Malectin	410	3	0.59	32.21
Q7Z7H5	TMED4	Transmembrane emp24 domain-containing protein 4	397	4	1.51	25.93
Q09328	MGAT5	Alpha-1,6-mannosylglycoprotein 6-beta-N-acetylglucosaminyltransferase A	386	1	0.06	84.49
Q96EE4	CCDC126	Coiled-coil domain-containing protein 126	385	1	0.29	15.66
Q9Y3P8	SIT1	Signaling threshold-regulating transmembrane adapter 1	360	1	0.23	21.11

P15153	RAC2	Ras-related C3 botulinum toxin substrate 2	347	3	2.16	21.42
P02042	HBD	Hemoglobin subunit delta	324	2	0.87	16.05
P43121	MCAM	Cell surface glycoprotein MUC18	312	3	0.28	71.56
P69905	HBA1	Hemoglobin subunit alpha	294	4	9.00	15.25
P25445	FAS	Tumor necrosis factor receptor superfamily member 6	280	3	0.41	37.71
P61009	SPCS3	Signal peptidase complex subunit 3	268	3	1.37	20.30
Q13291	SLAMF1	Signaling lymphocytic activation molecule	239	3	0.47	37.21
P12259	F5	Coagulation factor V	186	3	0.07	251.55
Q9HDC9	APMAP	Adipocyte plasma membrane-associated protein	182	5	0.59	46.45
P02649	APOE	Apolipoprotein E	162	2	0.20	36.13
P35443	THBS4	Thrombospondin-4	124	1	0.05	105.80
P28068	HLA-DMB	HLA class II histocompatibility antigen, DM beta chain	119	3	0.93	28.92
Q96PC5	MIA2	Melanoma inhibitory activity protein 2	95	3	0.08	159.74
O75063	FAM20B	Glycosaminoglycan xylosylkinase	84	1	0.30	46.40
Q5UCC4	EMC10	ER membrane protein complex subunit 10	63	2	0.43	27.33
Q9Y639	NPTN	Neuroplastin	49	2	0.21	44.36
Q96RQ9	IL4I1	L-amino-acid oxidase	36	2	0.22	62.84
Q96DU3	SLAMF6	SLAM family member 6	23	1	0.12	37.32
Q8WUT4	LRRN4	Leucine-rich repeat neuronal protein 4	17	1	0.07	78.79
Q96LC7	SIGLEC10	Sialic acid-binding Ig-like lectin 10	0	1	0.14	76.54

Table S3. Glycoproteins identified in BJAB cells with ≥ 5 abundance in Siglec-2-BA-Diaz@MNP **C** than Siglec-2-BA-Diaz-DSS@MNP

**Acc. No.**	**Gene**	**Description**	**Mascot Score**	**Unique Pep-tides**	**emP AI**	**Ratio: Siglec-2-BA-Diaz@MNP C / Siglec-2-BA-Diaz-DSS@MNP**
P20273	CD22	B-cell receptor CD22	11589	24	20.98	6.25
P01903	HLA-DRA	HLA class II histocompatibility antigen	10884	9	116.88	6.64
P20039	HLA-DRB1	HLA class II histocompatibility antigen	5652	1	41.17	6.31
P08195	SLC3A2	4F2 cell-surface antigen heavy chain	3147	18	3.15	7.37
P06576	ATP5B	ATP synthase subunit beta, mitochondrial	3079	21	10.60	22.86
P30443	HLA-A	HLA class I histocompatibility antigen, A-1 alpha chain	2097	4	5.70	6.55
P15391	CD19	B-lymphocyte antigen CD19	1567	8	1.22	5.00
P20036	HLA-DPA1	HLA class II histocompatibility antigen, DP alpha 1 chain	1414	5	10.94	6.00
Q13586	STIM1	Stromal interaction molecule 1	1322	15	2.57	10.77
P13473	LAMP2	Lysosome-associated membrane glycoprotein 2	1239	6	1.31	5.74
P04440	HLA-DPB1	HLA class II histocompatibility antigen, DP beta 1 chain	1195	5	2.73	8.25
P08575	PTPRC	Receptor-type tyrosine-protein phosphatase C	1134	18	0.94	10.89
P08238	HSP90AB1	Heat shock protein HSP 90-beta	1071	10	2.08	12.94
P02786	TFRC	Transferrin receptor protein 1	1021	9	0.50	5.57
P04222	HLA-C	HLA class I histocompatibility antigen, Cw-3 alpha chain	948	1	2.36	6.86
Q92542	NCSTN	Nicastrin	833	5	1.02	8.04
P04843	RPN1	Dolichyl-diphosphooligosaccharide--protein glycosyltransferase subunit 1	769	15	1.46	9.00
Q86UP2	KTN1	Kinectin	685	18	0.66	9.11

P14625	HSP90B1	Endoplasmin	659	7	0.50	7.74
P02748	C9	Complement component C9	592	2	0.25	13.00
P54709	ATP1B3	Sodium/potassium-transporting ATPase subunit beta-3	554	6	1.93	6.38
P09326	CD48	CD48 antigen	540	5	1.42	8.70
P62937	PPIA	Peptidyl-prolyl cis-trans isomerase A	523	5	1.28	5.29
P19440	GGT1	Gamma-glutamyltranspeptidase 1	509	3	0.33	5.64
P05538	HLA-DQB2	HLA class II histocompatibility antigen, DQ beta 2 chain	502	1	0.85	5.11
P61586	RHOA	Transforming protein RhoA	458	7	7.38	229.63
Q9BY67	CADM1	Cell adhesion molecule 1	448	3	0.47	7.06
P27797	CALR	Calreticulin	430	9	2.46	20.00
Q14165	MLEC	Malectin	410	3	0.59	17.14
P69905	HBA1	Hemoglobin subunit alpha	294	4	9.00	6.09
Q13291	SLAMF1	Signaling lymphocytic activation molecule	239	3	0.47	6.59
P02649	APOE	Apolipoprotein E	162	2	0.20	5.67
P28068	HLA-DMB	HLA class II histocompatibility antigen, DM beta chain	119	3	0.93	5.50
Q96PC5	MIA2	Melanoma inhibitory activity protein 2	95	3	0.08	5.00
Q96DU3	SLAMF6	SLAM family member 6	23	1	0.12	5.60
Q96LC7	SIGLEC10	Sialic acid-binding Ig-like lectin 10	0	1	0.14	5.42

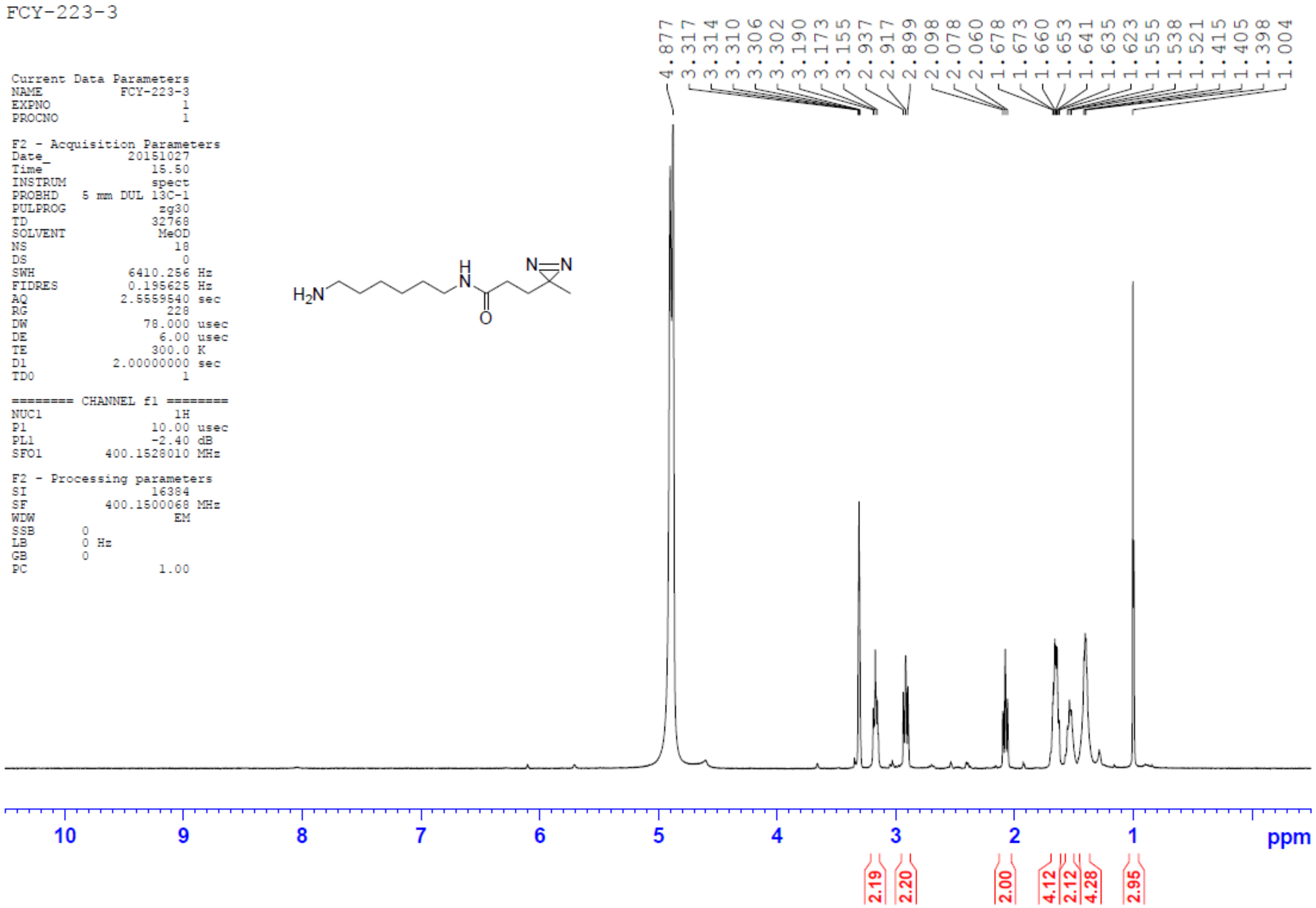
FCY-223-3
Current Data Parameters
NAME FCY-223-3
EXPNO 1
PROCNO 1
F2 - Acquisition Parameters
Date_ 20151027
Time 15.50
INSTRUM spect
PROBHD 5 mm DUL 13C-1
PULPROG zg30
TD 32768
SOLVENT MeOD
NS 18
DS 0
SWH 6410.256 Hz
FIDRES 0.195625 Hz
AQ 2.5559540 sec
RG 228
DW 78.000 usec
DE 6.00 usec
TE 300.0 K
D1 2.00000000 sec
TD0 1
======== CHANNEL f1 ========
NUC1 1H
P1 10.00 usec
PL1 -2.40 dB
SFO1 400.1528010 MHz
F2 - Processing parameters
SI 16384
SF 400.1500068 MHz
WDW EM
SSB 0
LB 0 Hz
GB 0
PC 1.00
H2N
H
N
O
N=N
4.877
3.317
3.314
3.310
3.306
3.302
3.190
3.173
3.155
2.937
2.917
2.899
2.098
2.078
2.060
1.678
1.673
1.660
1.653
1.641
1.635
1.623
1.555
1.538
1.521
1.415
1.405
1.398
1.004
10
9
8
7
6
5
4
3
2
1
ppm
2.19
2.20
2.00
4.12
2.12
4.28
2.95

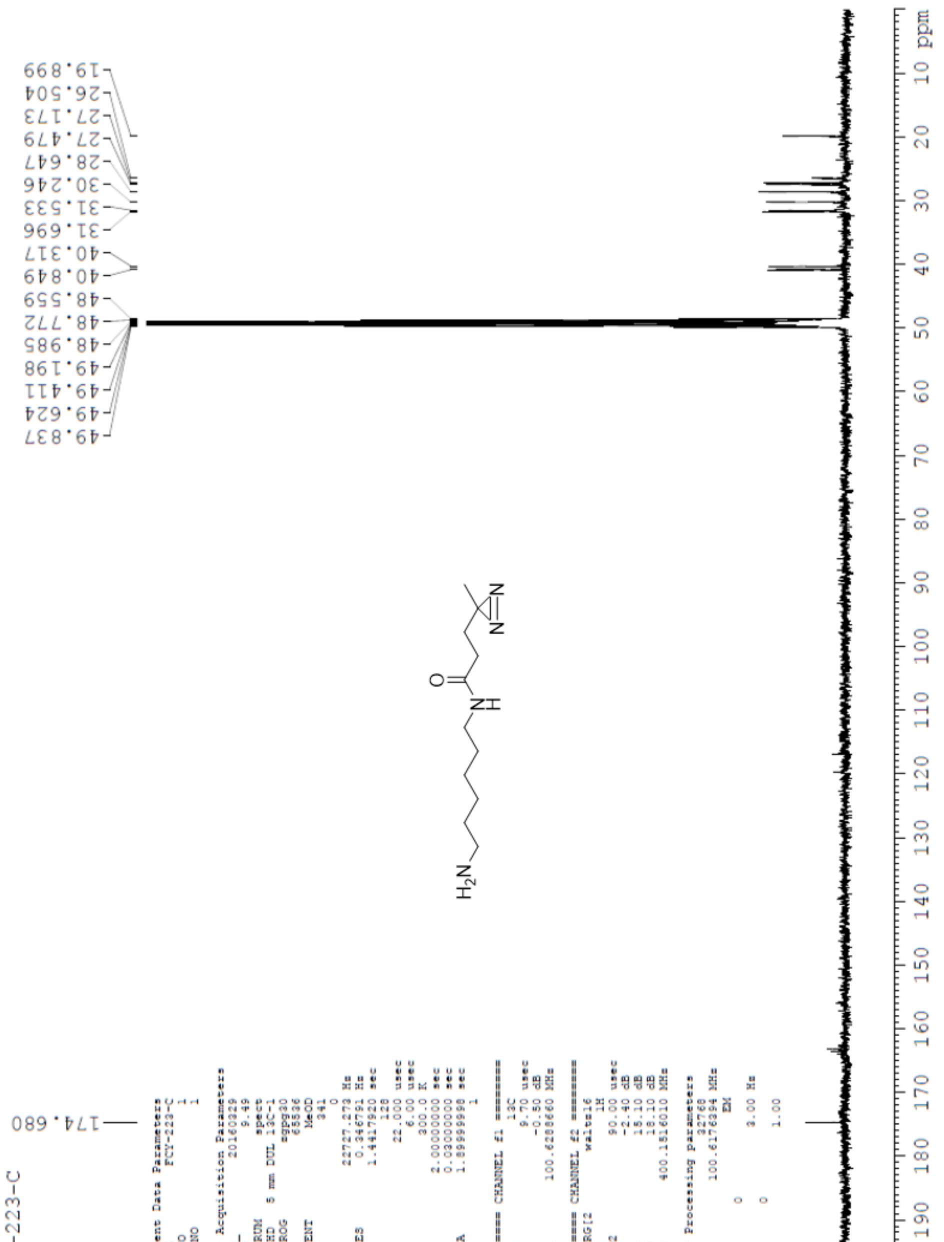
FCY-223-C
174.680
19.899
26.504
27.173
27.479
28.647
30.246
31.533
31.696
40.317
40.849
48.559
48.772
48.985
49.198
49.411
49.624
49.837
ppm

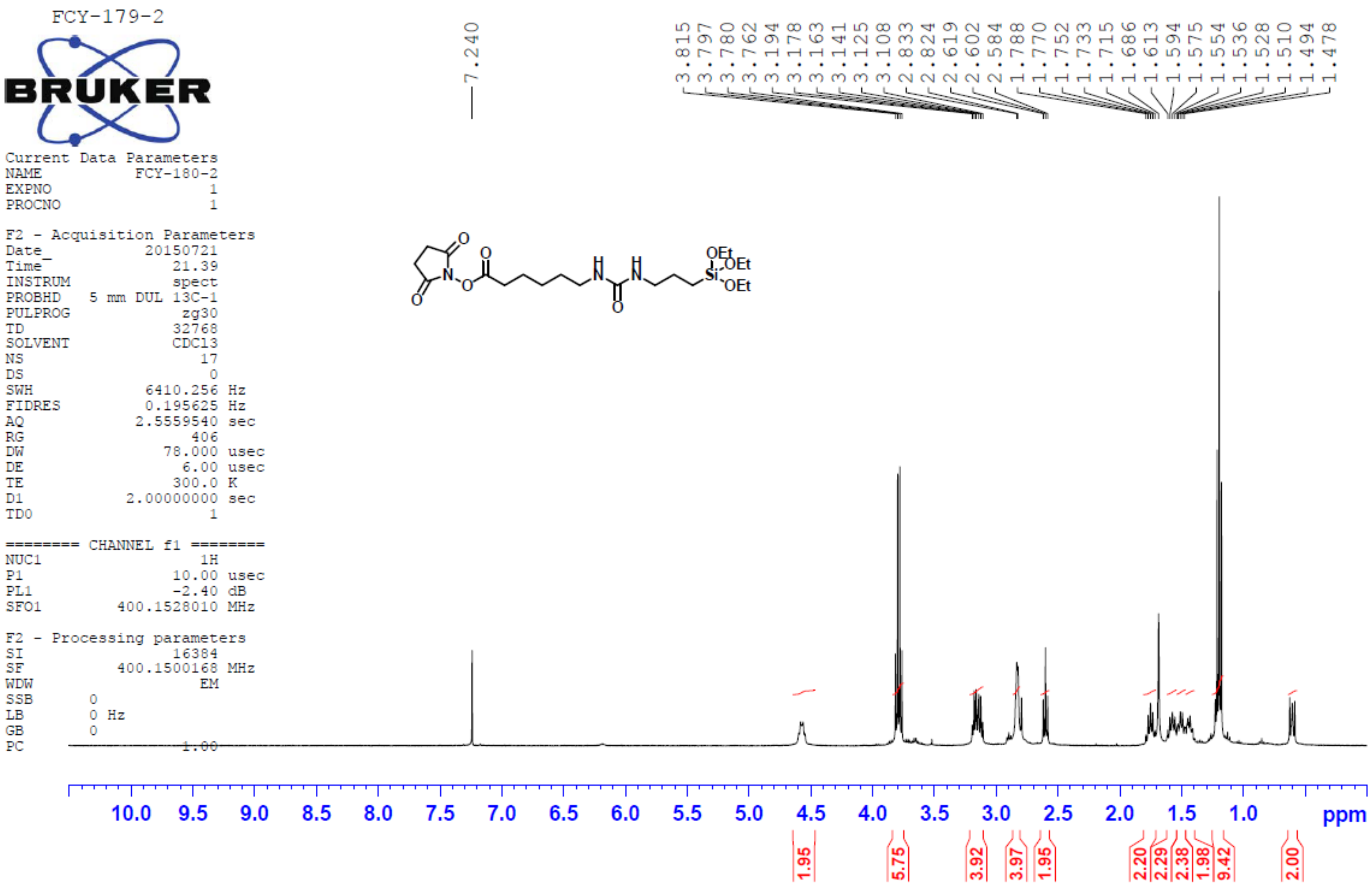
FCY-179-2
Current Data Parameters
NAME FCY-180-2
EXPNO 1
PROCNO 1
F2 - Acquisition Parameters
Date_ 20150721
Time 21.39
INSTRUM spect
PROBHD 5 mm DUL 13C-1
PULPROG zg30
TD 32768
SOLVENT CDCl3
NS 17
DS 0
SWH 6410.256 Hz
FIDRES 0.195625 Hz
AQ 2.5559540 sec
RG 406
DW 78.000 usec
DE 6.00 usec
TE 300.0 K
D1 2.00000000 sec
TD0 1
======== CHANNEL f1 ========
NUC1 1H
P1 10.00 usec
PL1 -2.40 dB
SFO1 400.1528010 MHz
F2 - Processing parameters
SI 16384
SF 400.1500168 MHz
WDW EM
SSB 0
LB 0 Hz
GB 0
PC 1.00
7.240
3.815 3.797 3.780 3.762 3.194 3.178 3.163 3.141 3.125 3.108 2.833 2.824 2.619 2.602 2.584 1.788 1.770 1.752 1.733 1.715 1.686 1.613 1.594 1.575 1.554 1.536 1.528 1.510 1.494 1.478
O
N
O
OEt
Si
OEt
OEt
H
N
10.0 9.5 9.0 8.5 8.0 7.5 7.0 6.5 6.0 5.5 5.0 4.5 4.0 3.5 3.0 2.5 2.0 1.5 1.0 ppm
1.95 5.75 3.92 3.97 1.95 2.20 2.29 2.38 1.98 9.42 2.00

FCY-180-C

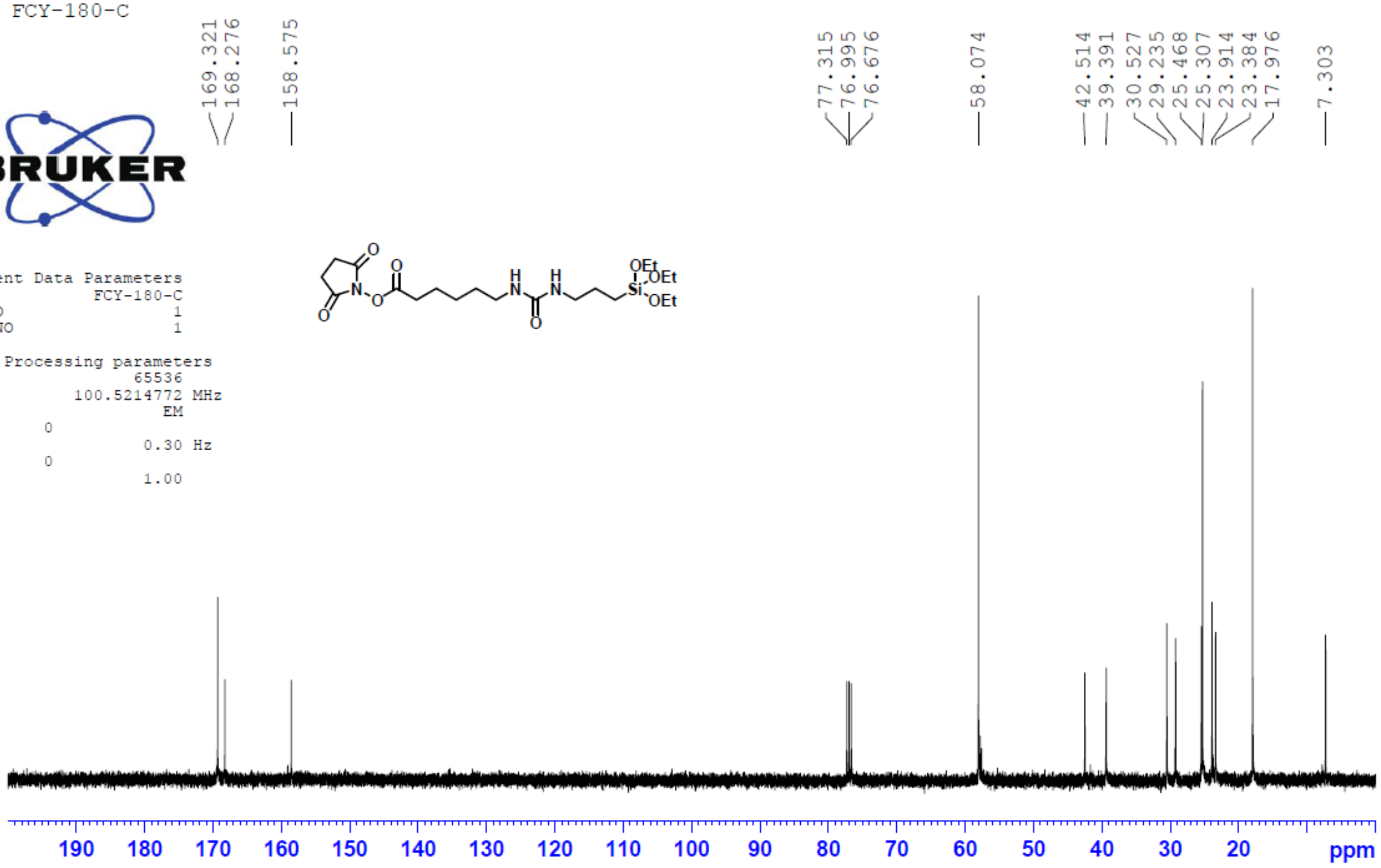


```
Current Data Parameters
NAME          FCY-180-C
EXPNO                 1
PROCNO                1

F2 - Processing parameters
SI                65536
SF         100.5214772 MHz
WDW                  EM
SSB       0
LB                 0.30 Hz
GB        0
PC                 1.00
```

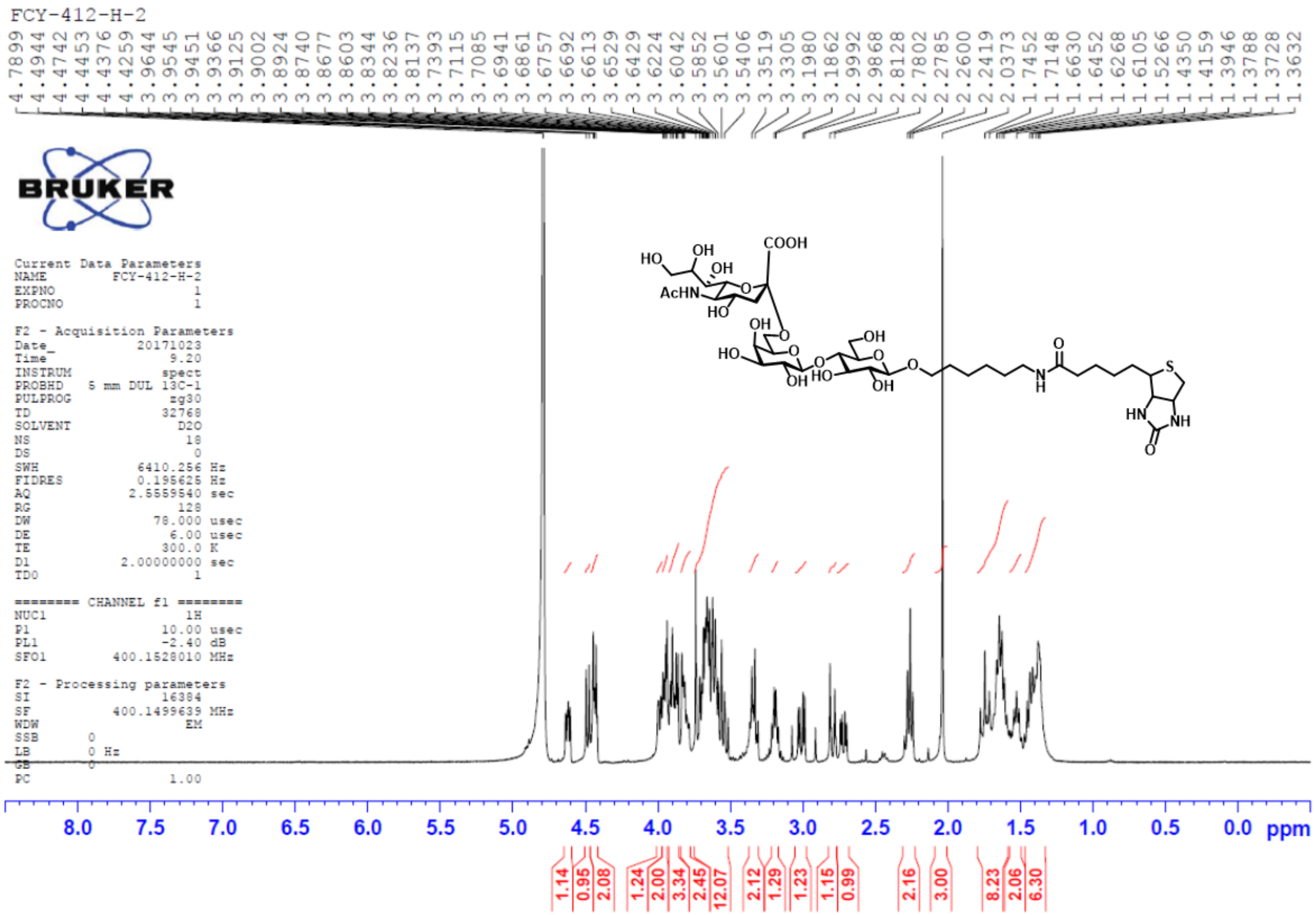
FCY-412-H-2
4.7899
4.4944
4.4742
4.4453
4.4376
4.4259
3.9644
3.9545
3.9451
3.9366
3.9125
3.9002
3.8924
3.8740
3.8677
3.8603
3.8344
3.8236
3.8137
3.7393
3.7115
3.7085
3.6941
3.6861
3.6757
3.6692
3.6613
3.6529
3.6429
3.6224
3.6042
3.5852
3.5601
3.5406
3.3519
3.3305
3.1980
3.1862
2.9992
2.9868
2.8128
2.7802
2.2785
2.2600
2.2419
2.0373
1.7452
1.7148
1.6630
1.6452
1.6268
1.6105
1.5266
1.4350
1.4159
1.3946
1.3788
1.3728
1.3632
BRUKER
Current Data Parameters
NAME FCY-412-H-2
EXPNO 1
PROCNO 1
F2 - Acquisition Parameters
Date_ 20171023
Time 9.20
INSTRUM spect
PROBHD 5 mm DUL 13C-1
PULPROG zg30
TD 32768
SOLVENT D2O
NS 18
DS 0
SWH 6410.256 Hz
FIDRES 0.195625 Hz
AQ 2.5559540 sec
RG 128
DW 78.000 usec
DE 6.00 usec
TE 300.0 K
D1 2.00000000 sec
TD0 1
======== CHANNEL f1 ========
NUC1 1H
P1 10.00 usec
PL1 -2.40 dB
SFO1 400.1528010 MHz
F2 - Processing parameters
SI 16384
SF 400.1499639 MHz
WDW EM
SSB 0
LB 0 Hz
GB 0
PC 1.00
HO
OH
OH
COOH
AcHN
HO
OH
O
HO
O
O
OH
O
OH
HO
OH
O
O
N
H
S
HN
NH
O
8.0
7.5
7.0
6.5
6.0
5.5
5.0
4.5
4.0
3.5
3.0
2.5
2.0
1.5
1.0
0.5
0.0
ppm
1.14
0.95
2.08
1.24
2.00
3.34
2.45
12.07
2.12
1.29
1.23
1.15
0.99
2.16
3.00
8.23
2.06
6.30

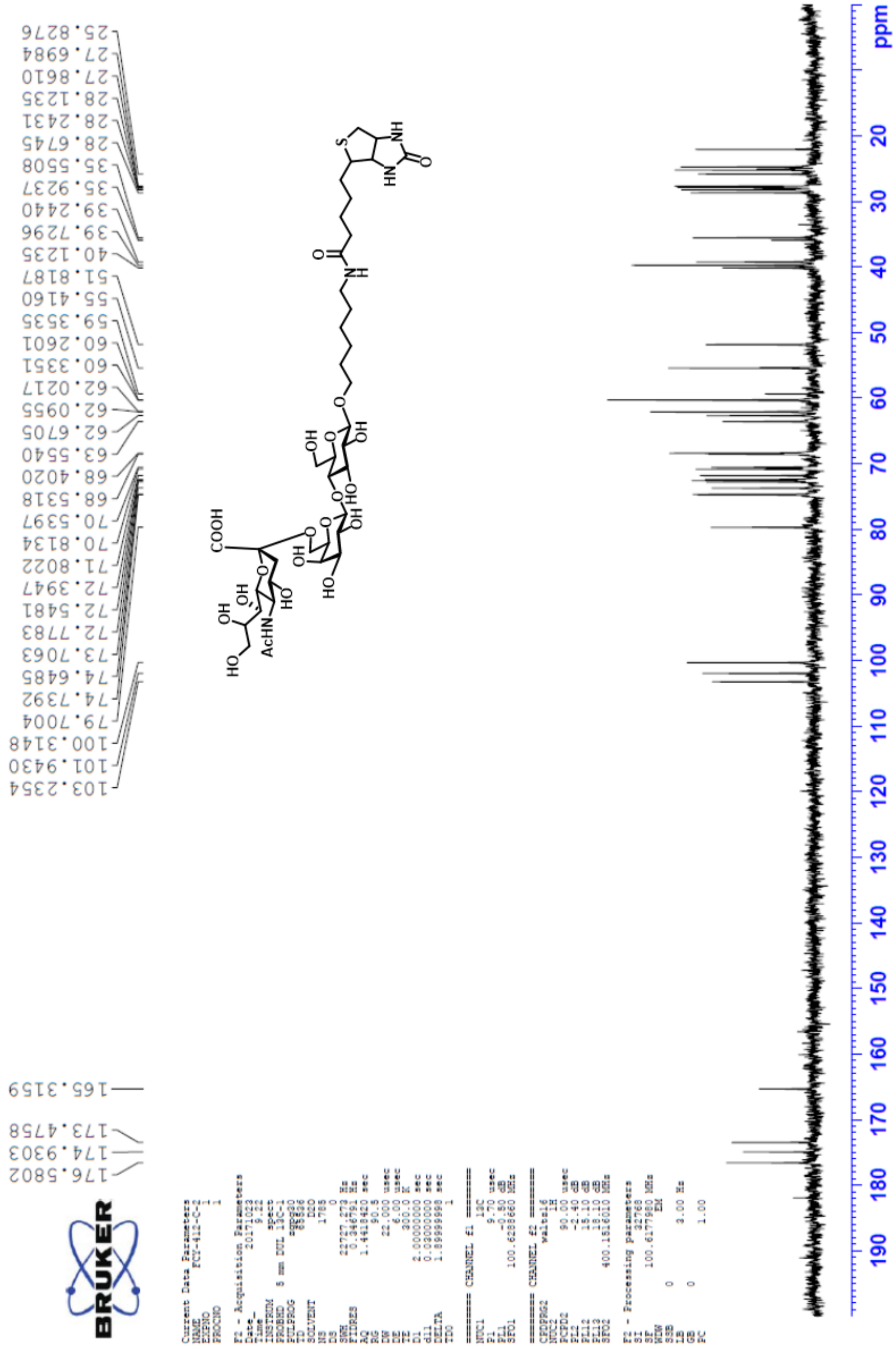

25.8276
27.6984
27.8610
28.1235
28.2431
28.6745
35.5508
35.9237
39.2440
39.7296
40.1235
51.8187
55.4160
59.3535
60.2601
60.3351
62.0217
62.0955
62.6705
63.5540
68.4020
68.5318
70.5397
70.8134
71.8022
72.3947
72.5481
72.7783
73.7063
74.6485
74.7392
79.7004
100.3148
101.9430
103.2354
165.3159
173.4758
174.9303
176.5802
ppm

Ref:

1. Tohru Kambe, Bruno E. Correia, Micah J. Niphakis, Benjamin F. Cravatt *J. Am. Chem. Soc.* **2014**, *136*, 10777−10782.
2. Hai Yu, Shengshu Huang, Harshal Chokhawala, Mingchi Sun, Haojie Zheng, Xi Chen *Angew. Chem. Int. Ed.* **2006**, *45*, 3938 –3944.
